# *Plasmodium vivax* Genetic Diversity in Panama: Challenges for Malaria Elimination in Mesoamerica

**DOI:** 10.3390/pathogens10080989

**Published:** 2021-08-05

**Authors:** Ana María Santamaría, Vanessa Vásquez, Chystrie Rigg, Franklyn Samudio, Dianik Moreno, Luis Romero, Azael Saldaña, Luis Fernando Chaves, José Eduardo Calzada

**Affiliations:** 1 Departamento de Investigación en Parasitología, Instituto Conmemorativo Gorgas de Estudios de la Salud, Panama 0816-02593, Panama; asantamaria@gorgas.gob.pa (A.M.S.); vvasquez@gorgas.gob.pa (V.V.); chrigg@gorgas.gob.pa (C.R.); fsamudio@gorgas.gob.pa (F.S.); asaldana@gorgas.gob.pa (A.S.); 2 Facultad de Medicina y Medicina Veterinaria, Universidad de Panamá, Panama 0816-03366, Panama; 3 Laboratorio Central de Referencia en Salud Publica, Instituto Conmemorativo Gorgas de Estudios de la Salud, Panama 0816-02593, Panama; dmoreno@gorgas.gob.pa (D.M.); lromero@gorgas.gob.pa (L.R.); 4 Investigador Asociado, Instituto Conmemorativo Gorgas de Estudios de la Salud, Panama 0816-02593, Panama

**Keywords:** *Plasmodium vivax*, genotype, molecular epidemiology, elimination, Panama, Mesoamerica

## Abstract

Panama and all nations within the Mesoamerican region have committed to eliminate malaria within this decade. With more than 90% of the malaria cases in this region caused by *Plasmodium vivax*, an efficient national/regional elimination plan must include a comprehensive study of this parasite’s genetic diversity. Here, we retrospectively analyzed *P. vivax* genetic diversity in autochthonous and imported field isolates collected in different endemic regions in Panama from 2007 to 2020, using highly polymorphic markers (*csp*, *msp*-1, and *msp*-3α). We did the analysis using molecular techniques that are cost-effective for malaria molecular surveillance within Mesoamerica. Thus, we used molecular analyses that are feasible for malaria molecular surveillance within the region, and that can provide useful information for policy and decision making about malaria elimination. We also evaluated if haplotypes established by combining the genotypes found in these genes were associated with relevant epidemiological variables and showed structure across the transmission foci that have been observed in Panama. Ten different haplotypes were identified, some of them strongly associated with geographical origin, age, and collection year. Phylogenetic analysis of *csp* (central repeat domain) revealed that both major variant types (vk210 and vk247) were circulating in Panama. Variant vk247 was restricted to the eastern endemic regions, while vk210 was predominant (77.3%) and widespread, displaying higher diversity (14 alleles) and geographically biased alleles. The regional implications of these molecular findings for the control of *P. vivax* malaria to achieve elimination across Mesoamerica are discussed.

## 1. Introduction

**Figure 1:**
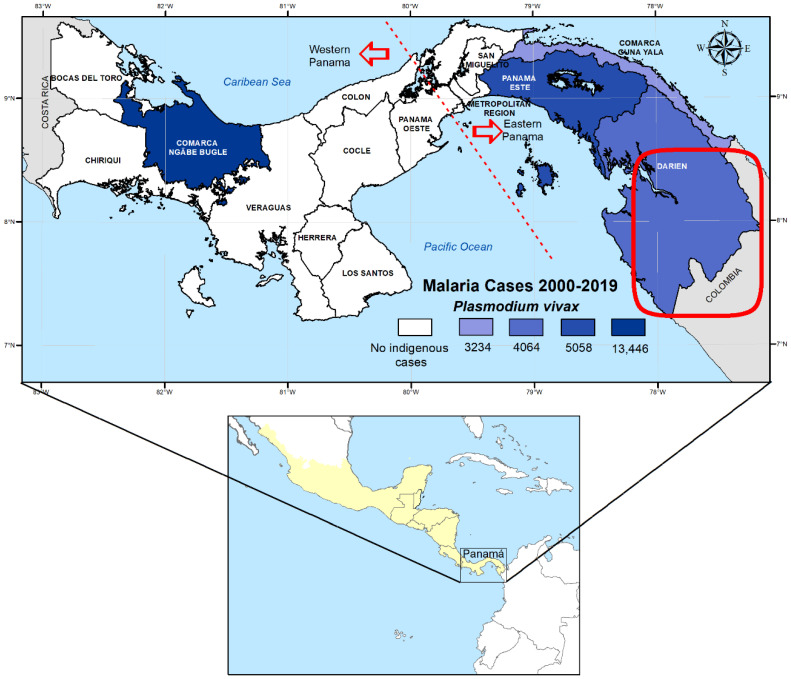
Map of the Panama showing health regions with active *Plasmodium vivax* transmission based on the cumulative number of cases between 2000 and 2019. The dashed lines in the map indicate the Panama Canal pathway that artificially divides the country into eastern and western Panama. The red circle represents the location of the Darien Gap consisting of a road-less swath of swampland and rainforest within Panama’s Darién Province and the northern portion of Colombia’s Chocó Department. The Darien Gap is a corridor used by migrants from around the globe intending to reach the United States or Canada. The lower inset map shows the location of Panama within the Mesoamerican region, colored in yellow.

Given the relatively low number of malaria cases and deaths, and its strategic geographical position as a corridor connecting South and North America (Figure 1); Mesoamerica has been recently targeted by the World Health Organization (WHO) as a tactical subregion with malaria elimination potential in the short term [1,2]. In fact, less than 1% of the global malaria burden occurs in this subregion, which is mostly (around 95%) caused by *Plasmodium vivax* parasites [3]. From the nine countries comprising Mesoamerica (southeastern Mexico and the countries of Central America, including Panama) (Figure 1), El Salvador was the first country in Central America to be recently certified malaria-free by WHO, and three other countries (Mexico, Belize, and Costa Rica) were identified with the potential to achieve zero indigenous cases of malaria by 2021 [4,5]. However, despite the favorable epidemiological conditions in the region, many of these countries have not been able to meet the elimination goals set by WHO [1,3], facing important technical, administrative, and financial challenges that have been identified, but not properly addressed by heath authorities [6,7,8]. 

Panama, located at the southern end of Mesoamerica (Figure 1), has also regionally committed to eliminate malaria by 2021 [9]. During the past years, however, a relentless malaria burden has been observed in recognized endemic areas; a situation further complicated by the reestablishment of *P. falciparum* transmission in the eastern region of the country [10]. Moreover, indigenous populations account for more than 90% of local malaria cases [10,11]. Most of these indigenous populations (92%) live in extreme poverty, with significant higher rates of malnutrition, and infant and maternal mortality, especially when compared with the rest of the Panamanian population [9,10,11,12,13]. Panama is also a hotspot for the transit of economic migrants worldwide [14]. Recent studies have shown a growing number of imported malaria continuously entering from South America through the Darién Gap, an area across the Colombia and Panama border (Figure 1) [10,15]. *Plasmodium* parasites isolated from these imported cases have shown a higher genetic diversity when compared with the genetic structure displayed by local cases [15]. 

The majority (95%) of the malaria cases registered in Panama since 2000 have been caused by *P. vivax* [10]. This fact suggests that an efficient elimination plan must include a comprehensive study of *P. vivax* biology considering the species peculiarities that hamper its control. In this sense, knowing the genetic structure of *P. vivax* circulating in the country over years can elucidate not only the origins and connectivity of the transmission, but also evidence the association of certain genotypes with the pathogenesis and epidemiology of the disease. This information is crucial to design appropriate local and regional control measures, which within the Mesoamerican context also must be cost-effective, given that all nations in the region are either middle- or low-income countries. In Mesoamerica, different molecular strategies have been designed to explore *P. vivax* diversity, including single locus typing, microsatellite analysis, molecular DNA barcodes, and deep sequencing approaches [16]. Of these, exploiting the single nucleotide and length polymorphisms observed in the circumsporozoite protein (*csp*) and the merozoite surface proteins (*msp*-1 and *msp*-3α) of genes have been the most common and widely used approaches to assess and compare the genotypes of *P. vivax* infecting humans and vectors [16]. Moreover, the molecular typification of surveillance samples with these tools is feasible and cost-effective within the Mesoamerican context where thermocyclers and Sanger sequencing can be implemented by most national reference laboratories in the region.

So far, few studies have explored the genetic diversity of *P. vivax* parasites circulating in countries from Mesoamerica. Here, we retrospectively analyzed *P. vivax* genetic diversity in autochthonous and imported field isolates collected in different endemic regions in Panama from 2007 to 2020, using the highly polymorphic markers (*csp*, *msp*-1, and *msp*-3α). We then evaluated if haplotypes established by combining the genotypes found in these genes were associated with demographic, epidemiological, and geographical parameters relevant to malaria infection. The regional implications of these molecular findings for the control of *P. vivax* malaria to attain the shared elimination goal are discussed.

## 2. Results

A sample of 304 *P. vivax* isolates, confirmed by PCR, and collected between 2007 and 2020, were included in the study. Two mixed infections with *P. falciparum* were detected but were not further included in the genotyping analysis. The patients came from all the recognized endemic regions in the country located west (Veraguas and Bocas del Toro) and east (Panamá Este, Guna Yala, and Darién) of the Panama Canal (Figure 1). Eighteen samples were captured from patients in health facilities located in the non-endemic metropolitan region of Panama (Figure 1). However, the identification of a precise geographical origin in most of these infections was not feasible based on the travel history declared by the patient. In addition, 40 samples were collected from patients that came from different endemic countries as declared by their travel history: 26 from South America, 5 from Central America, 4 from Asia, and 4 from Africa. One patient originated from Germany. However, at the time of sample collection and diagnosis, the patient was visiting the western part of the Veraguas region. Thus, it was considered to be a local infection. 

### 2.1. Genotyping of Csp, Msp-1, and Msp-3α Genes by PCR-RFLP

From the 304 samples initially included in this study, 208 (68.4%) were successfully amplified and genotyped for *csp*, 234 (76.9%) for *msp*-1, and 236 (77.6%) for *msp*-3α (Table 1). Due to the variability in sample amplification, it was possible to genotype 163 (53.66%) samples combining the data gathered on the three genes.

Restriction profile analysis of the *csp* gene revealed that two described types of *P. vivax* repeats (vk210 and vk247) were present in the studied samples, but with different proportions (vk210 81.7% vs. vk247 18.3%) and with distributions that generally correspond to their geographical origin (Table 1 and Figure S1). For instance, all parasites from endemic regions located in western Panama (Bocas del Toro and Veraguas) were single infections of the vk210 type. The vk210 type was also frequently detected in eastern endemic regions (Panamá Este, Guna Yala, and Darién). Interestingly, an important number of samples (34/208; 16.3%) from eastern regions located near the border with Colombia (Guna Yala and Darién) harbored the vk247 type (Table 1). In the 16 suspected imported cases genotyped for this gene, both repeat types were observed, with predominance of the vk210 variant (12 vk210 vs. 4 vk247). No mixed infections of both repeat types were observed in any of the samples analyzed. In our study, it was relatively subjective to further interpret the restriction fragments generated in the *csp*-RFLP analysis by our visual detection method. Thus, the assignment of possible allelic variants within vk210 and vk247 types was further performed by sequencing analysis of the *csp*-amplified product as described in the Section 4.

Four different alleles (coded as Genotypes I–IV) were identified for the *msp*-1 gene after RFLP analysis with *Alu* I enzyme (Table 1 and Figure S1). Genotype I was predominant (177/234; 75.6%) and evenly distributed in all endemic regions, including the imported cases. Genotype II was found at a lower proportion (54/234; 23.1%) at endemic regions in both sides of the country, except for Veraguas. While Genotypes III and IV were only detected in three imported cases from Asia (Genotype III: one sample from China and Genotype IV: two samples from India).

Three different patterns (assigned Genotypes I–III) were observed when the *msp*-3 α-amplified product was digested with the *Hha* I enzyme (Table 1 and Figure S1). Genotype I was predominant (191/236; 80.1%) and identified in all endemic regions and in imported cases. Genotype II was found at a lower frequency (43/236; 18.2%) and was absent in endemic regions located west of the Panama Canal. Genotype III was only found in two samples, one from the eastern Darién region and one imported from China.

**Figure 2:**
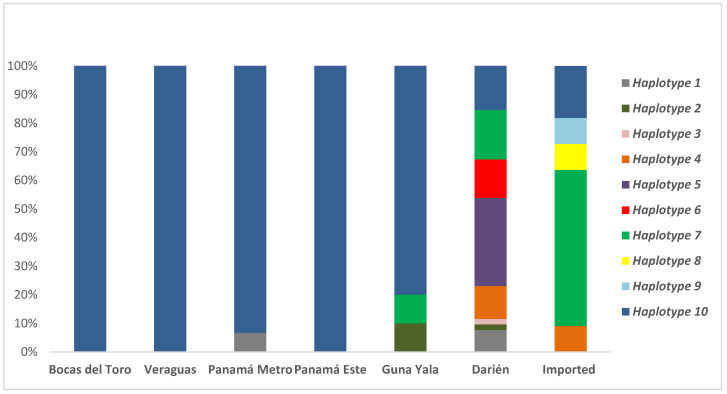
Haplotype frequency and distribution inferred by combining the alleles detected in the three loci (*csp*, *msp-1,* and *msp-3*α) in *Plasmodium vivax* indigenous and assumed imported cases based on travel history.

Combined analysis of the three genetic markers allowed for the identification of 10 different haplotypes (termed Haplotypes 1–10) in the 163 samples evaluated (Figure 2 and Table 2). Haplotype 10 was predominant (107/163; 65.6%) and widespread in samples from all eastern and western endemic regions, including imported samples and cases reported in the non-endemic metropolitan region. Haplotypes H1–H7 were found at lower frequencies and only in regions located to the east of the Panama Canal and imported cases. Interestingly, haplotypes H3 (0.6%), H5 (9.8%), and H6 (4.3%) were exclusively found in samples from the Darién region. Similarly, haplotype H1 was found in four samples from Darién (2.4%) and in one sample (0.6%) collected from a patient in the non-endemic region of Panamá Metro from which the origin of the infection was not possible to verify based solely on the travel history. Haplotypes H8 and H9 were only found in two imported cases, one from China and one from India, respectively (Table 2).

In general, local samples collected in eastern endemic regions (Guna Yala and Darién) presented a higher diversity compared to the homogeneity observed in samples from the western areas (Bocas del Toro and Veraguas), with some haplotypes geographically restricted to the Darién endemic region closer to the Colombian border (Figure 2).

### 2.2. Multiple Correspondence Analysis

**Figure 3:**
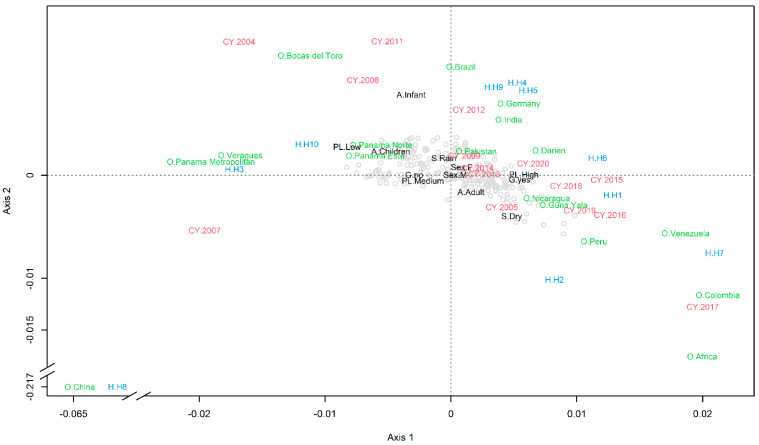
Multiple correspondence analysis. In the plot, the coordinates for each studied subject are presented by gray circles, and the dashed lines cross at the origin of the plot—the point where variables are independent. Text indicates the centroids for the different categories of the studies variables which included the following: in blue are the Haplotypes (H1 to H10); in red is the collection year (CY); in green is the origin (O); and the rest of the variables are in black, which included the following: age (A: Preschool children, Infant, Adult); sex (M: Male, F: Female), season (S: Rainy, Dry), gametocyte presence (G: Yes or No), parasite load (PL: High, Medium, Low). The correlation for Axis 1 was 0.533 and for Axis 2 was 0.507, with both axes explaining up to 14.86% of the variance in the data. To ease visualization, Axis 2 has coordinates of H8, A. Children and sex F., were changed by respectively adding the following values: −0.00045, −0.00070, and −0.00045. Axis 1 and Axis 2 have breaks to ease the visualization of H8 and O from China.

We performed a multiple correspondence analysis (MCA) to study the association between the haplotypes, demographic data from the patients (including sex and age), and data about the sampling season, year, the geographic origin of the sample, parasitic load, and gametocyte presence. A detailed explanation of this method and its assumptions is presented in the Section 4 at the end of this article. The MCA showed that samples from any sex, the rainy seasons, and from the years 2009, 2013, and 2014 were not strongly associated with any other variable, as they were near the origin of the plot, i.e., coordinates (0, 0) (Figure 3). High parasitemia was associated with gametocyte presence and medium parasitemia with gametocyte absence, as indicated by the distance between the coordinates for such variables in Figure 3. Haplotype 8 and Chinese origin were strongly associated, as shown by the short distance between these two variables in the lower left corner of Figure 3. The MCA also suggests that haplotypes 1, 2, 4, 5, 6, 7, and 9 were associated with imported cases and with cases observed in the eastern Panamanian provinces of Darién and Guna Yala, for cases occurring in adults in 2005 and from 2012 to 2020, as shown on the positive side of Axis 1 of Figure 3. Meanwhile haplotypes 3 and 10 were exclusively found in the western Panamanian provinces of Panama (Este, Norte, and Metropolitan), as well as Veraguas and Bocas del Toro, in samples from infants and children collected in 2004, 2008, and 2011, as can be observed on the negative side of Axis 1 of Figure 3.

### 2.3. Csp Sequencing and Phylogenetic Analysis

**Figure 4:**
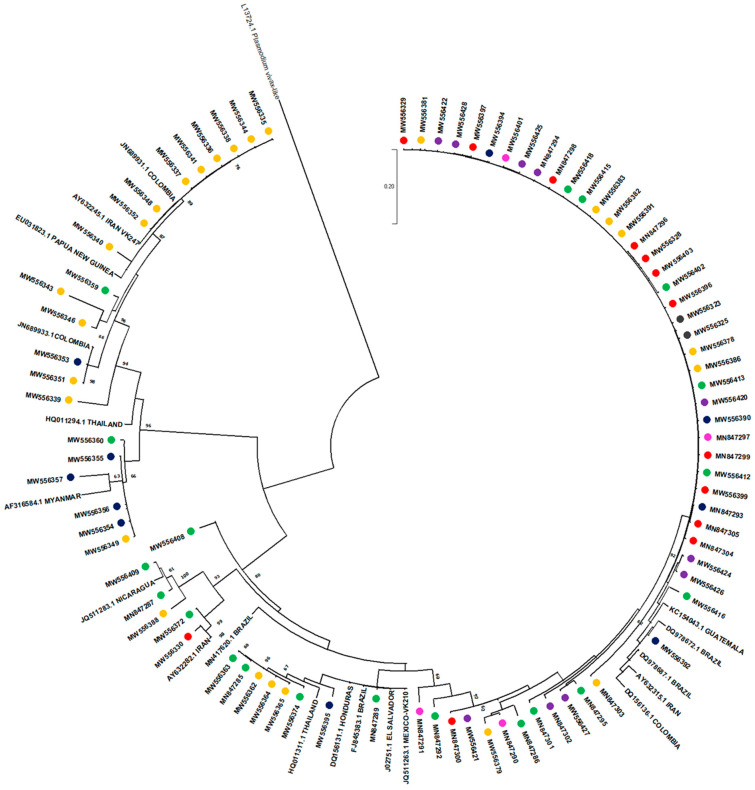
Phylogenetic tree constructed from the *Plasmodium vivax csp* gene central region nucleotide sequences. Sequences from this study are in colored circles: green are imported samples (20), red are from Panamá Este (13), yellow from Darién (26), blue from Guna Yala (10), pink from Panamá Metro (4), violet from Veraguas (10), and grey from Bocas del Toro (2) (accession numbers MW556323-MW556428 and MW847285-MW847305). The evolutionary history was inferred with the maximum likelihood method and Tamura-Nei model. The tree with the highest log likelihood (−7501.50) is shown. This analysis involved 105 nucleotide sequences, 85 are samples of this study and 20 are GenBank references. Evolutionary analyses were conducted in MEGA X with 1000 replications. Reference sequences are identified with their accession numbers and geographical location of parasite isolates. The tree was rooted using the reference sequence (L13724.1) of *Plasmodium vivax*-like *csp* as the out-group.

Based on the quality of the sequences, 85 isolates from this study (accession numbers MW556323–MW556428 and MN847285-MN847305) were selected for direct sequence analysis on amplified *csp* products: 20 imported and 65 indigenous isolates (2 Bocas del Toro, 10 Veraguas, 4 Metropolitan area, 13 Panamá Este, 10 Guna Yala, and 26 Darién). A phylogeny tree was estimated using the maximum likelihood method. The final phylogenetic analysis contained 105 nucleotide sequences, including for comparison 20 GenBank reference sequences collected from different endemic regions around the world (Figure 4). Complete nucleotides alignment is available upon request.

The sequences from this study clearly grouped into two distinct clades that corresponded to the *csp* vk210 and vk247 types (Figure 4). In each clade, several subdivisions were observed for both types. In agreement with the haplotype analysis, no local samples collected from the western side of the country clustered within the vk247 clade. Six samples from this study (three from Guna Yala (MW556354–MW556357), one from Darién (MW556349), and one imported from Perú in South America (MW556360)) showed higher genetic relatedness, clustering in a distinct subclade within the vk247 clade. The vk210 clade contained isolates from both sides of the country, as well as imported cases. No clear geographic clustering was observed in this clade among indigenous or imported samples when compared with the reference sequences evaluated from different latitudes.

### 2.4. Analysis of Csp Allelic Variants

The central repeat domain sequence of the *csp* gene was analyzed in 85 isolates. The aligned nucleotide sequences were converted into amino acids, evidencing the two major types of nonapeptides repeats distinctive of the vk210 and vk247 allelic types. No mixed infections with these two different allelic types, nor with the *P. vivax*-like variant, were detected. The vk210 variants were predominant (64/85; 77.3%) and were present in all endemic regions and in imported cases, while the vk247-type variant was only observed in 19 (21%) samples from the eastern provinces (Darién and Guna Yala) and in two imported isolates: one from China (MW556359) and the other from Perú (MW556360).

**Figure 5:**
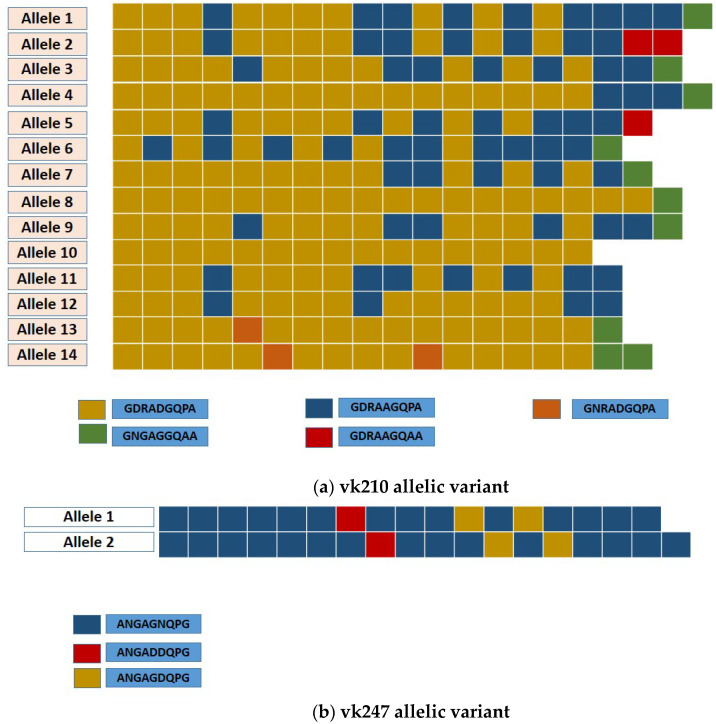
Schematic representation of the number, type, and combination of nonapeptides of the *Plasmodium vivax csp* gene observed in the central region in the different alleles identified in this study: (**a**) vk210 variant and (**b**) vk247 variant. Each color represents one of the nonapeptide motifs found for each *csp* variant.

Based on the number of repeats and thr arrangement of the nonapeptides variants in both allelic types, 16 different alleles for the *csp* gene were identified in this study: 2 in vk247 and 14 in vk210 (Figure 5). Within the vk247 samples, three repeats were observed in all isolates (ANGAGNQPG/ANGAGDQPG/ANGADDQPG) with only two repeat numbers (17 or 18 units) (Figure 5). Allele 1 was predominant and found in 71.4% (15/21) of the vk247 analyzed samples, mostly from Darién province. A higher diversity was observed for the vk210 type, as polymorphisms were observed not only in the number of the nonapeptide repeats (17–20 times), but also on the frequency and arrangement of variants in the repeat unit (Table 3 and Figure 5). Besides the common nonapeptides that define the vk210 allelic type (GDRA(D/A)GQPA), three other nonapeptide variants were identified, two of which were found solely in imported isolates and one of them in two local samples from Darién and one imported from Venezuela (MN847287) (Table 3 and Figure 5). Among the 14 vk210 alleles identified, allele 3 was the most common and widely distributed along the endemic regions of the country, accounting for 65.6% (42/64) of the vk210 isolates. The frequency of the remaining alleles varied from one (1.6%) to five (7.8%). Six (9.4%) vk210 alleles were exclusively found in imported cases, five (7.8%) in local cases, and three (4.7%) were identified in both local and imported cases (Table 3 and Figure 5).

An uneven distribution of certain vk210 alleles was observed between the different endemic regions in Panama. For instance, alleles 1, 6, and 14 were restricted to specific Panamanian regions in the east, while alleles 11 and 12 were limited to the west of the country. Moreover, alleles 2, 5, 7, 8, 10, and 13 were exclusively detected in imported isolates (Table 3). It is possible, however, that the absence or low frequency observed for some alleles may be due to different sampling number among the studied locations. Nevertheless, this information is important to trace the origin of *P. vivax* infections, particularly from samples collected in non-endemic areas of the country, like Panamá Metro, whose origin is sometimes difficult to determine based only on the travel history declared by the patient. 

When further comparing *csp* sequences obtained from eastern (Darién, Guna Yala, and Panamá Este) Panama with those collected in the western side of the country (Veraguas and Bocas del Toro) and with imported cases, a possible geographical bias of certain the alleles was observed (Table 3 and Figure 4). First, vk247 was not detected in isolates from the western region of the country. Regarding vk210, four allelic variants were circulating in the western side of the country while six were observed in the eastern side, with only two vk210 variants circulating in both sides (Table 3 and Figure 5). Isolates from imported cases exhibit a higher diversity consistent with their different geographic origin, displaying 6 alleles (42.82%) out of the 14 vk210 alleles identified in this study.

## 3. Discussion

Panama is narrow strip of land strategically located at the southeastern end of Mesoamerica joining the continents of North and South America (Figure 1). Despite its relatively small area (75,417 km^2^) and population (~4 M), Panama exhibits a high biological diversity with a great variety of landscapes and habitats and diverse indigenous territories (comarcas) inhabited by different ethnic groups that together represent around 12% of the total population [17]. These factors have strongly influenced malaria epidemiology over the years in the country, showing during the last decade a marked endemicity in specific areas located at the eastern and western ends of Panama (Figure 1), mostly inhabited by these indigenous populations [10]. In general, Panama is considered a low malaria transmission country. However, in the last two decades the country has experienced a sustained malaria burden averaging 1460 cases, most cases (90%) due to infections by *P. vivax* [10]. Despite Panama’s inability to meet previous malaria transmission reduction goals set by WHO [3], the country has regionally committed to eliminate malaria by 2021. In this context, we explored the genetic diversity of local and imported *P. vivax* cases to provide useful information that can be integrated in the national elimination plan as an indicator of the transmission intensity in the different endemic areas. Furthermore, this information will be valuable to assess the origin of infections, the parasite population complexity, and ultimately as a measure of the potential resilience of *P. vivax* populations to interventions [18]. 

With this purpose, three polymorphic loci in *P. vivax* were evaluated as follows: the repeat region of *csp*, the F2 fragment of *msp*-1m and the alpha segment of *msp*-3. Individually, *msp*-3 was the less informative in our study, as only three genotypes were detected with this marker. However, this may be an underestimation of the real diversity, as the PCR-RFLP approach, and particularly the visual detection method that we used to distinguish alleles in classical gel electrophoresis, have a low discrimination power that can add a degree of subjectivity to the analysis [19]. This fact should be considered when selecting individual markers and genotyping approaches, and especially when comparing results between Mesoamerican countries, since the diversity of a given marker and the number of markers required to accurately genotype *P. vivax* infections will depend not only on different approaches and detection methods, but also may differ between geographical locations due to variations in local malaria epidemiology, that might render noncomparable results [20].

Nonetheless, when evaluating the genotyping results obtained with individual genes (Table 1) and particularly when combining information of the three markers (Table 2), it was evident the genetic homogeneity exhibited through time by local *P. vivax* parasites circulating in western Panama, contrasting with the high diversity displayed by the ones collected in eastern Panama, particularly from the Darién region that is closer to the Colombian border (Table 2 and Figure 2). As expected, due to their different geographical origin, isolates from imported cases also exhibited a high diversity.

In general, our genotyping results coupled with the MCA analysis evidenced a strong association of certain local haplotypes with their geographical origin, and with important epidemiological variables such as the age of the patients and collection year of the samples (Figure 3). Altogether, this baseline genetic information is useful to develop effective methods to trace the origin of infections and to measure the impact of local interventions towards the elimination goal. This is especially important in settings where it is assumed that most cases are imported and that local outbreaks are triggered by initial imported infections. This, for example, is the situation in Costa Rica where recent outbreaks have been linked with migrant workers, but no evidence grounded on genetic markers have been used to support the inference [21].

A closer analysis of the *csp* sequences suggest that the *P. vivax* parasites circulating in the eastern side of the country were genetically dissimilar and exhibit a higher diversity than those collected in the western side (Table 3 and Figure 5). For instance, the vk247 variant was only observed in the eastern side, and more specifically in the two endemic regions closer to the Colombian border (Guna Yala and Darién). Interestingly, these eastern regions are also endemic for *P. falciparum* transmission and both are frequently used as arrival points by extracontinental economic migrants from various malaria-endemic regions heading to the United States [22,23]. Similar with our findings, vk247 is not the dominant type in local human isolates found in the rest of the Mesoamerican countries located to the west of the Panama Canal. In fact, in this region, vk247 has only been so far described in symptomatic patients from southern Mexico [24,25], but has not been detected in local isolates from Nicaragua [24], Honduras [26], or Guatemala [27]. However, vk247 is the prevalent phenotype in several endemic localities located in western Colombia [28,29], many of which are pathways used by extracontinental migrants before entering Panama through the permeable Panama/Colombia border in their way to the U.S. (Figure 1) [24]. As observed in other endemic regions, vk210 was the dominant *csp* variant in all studied areas of Panama, also displaying a higher diversity than vk247 (2 alleles in vk247 vs. 14 alleles in vk210). Interestingly, a geographical bias in the distribution of the 14 vk210, alleles detected in local isolates was also observed, with some alleles (alleles 11 and 12) being unique to the western side, while alleles 1, 4, 6, and 14 were found exclusively in the eastern side (Table 3). Our results also showed, however, that endemic regions from both sides of the country shared similar haplotypes and *csp* vk210 alleles. It is possible that these genetically similar parasites have the same founder origin despite the spatial distance between these indigenous autonomous areas and the unlikely mobility of the infected native population between different ethnic reservations located on both sides of the country (Figure 1).

*Plasmodium* genetic diversity is strongly influenced by several epidemiological factors, including transmission intensity, vector prevalence, host genetics, and a variety of environmental variables [16,18,30]. Although Panama has a small territory, ecological conditions are significantly different between the eastern and western endemic sides of the country, particularly in the amount and seasonal distribution of rainfall, mean annual temperatures, and daily fluctuations in temperature and in land cover [17]. According to the Köppen climate classification, malaria-endemic areas in the west have a tropical rainforest climate (Afi), while most endemic regions in the east have a tropical savanna climate (Awi) [17]. It is possible that these environmental and climate conditions have influenced local malaria transmission and consequently impacted the *Plasmodium* genetic structure observed in these regions of Panama.

Malaria is unevenly distributed among the Panamanian population. In fact, a disproportionate number of cases (over 85% of the total malaria cases during the past 40 years) occur in ethnic minorities that inhabit autonomous regions (Comarcas) of Panama. In general, these indigenous groups are socially marginalized and vulnerable populations. In these populations the use of conventional tools for malaria control have been less effective. The failure to control malaria in these areas is not only due to the social and health inequalities that affect these minorities, but also because of a lack of intercultural understanding, and other factors such as geographical isolation, internal movement across the country, and cross-border movement, particularly between Colombia and the eastern indigenous territories of Panama. 

There are marked differences between the ethnic groups that occupy the “comarcas” in both sides of the country in terms of cultural practices, languages, and lifestyles that are of relevance to malaria risk. The eastern comarcas are closer to Colombia and are mainly inhabited by the Gunas and Embera-Wounan ethnic groups. There is a frequent movement of the Gunas across their homeland, which comprises certain malaria hotspots in northern Colombia where human mobility has been shown to play a major role in malaria persistence. The Embera-Wounan comarca is next to the border with Colombia, and waves of refugees and economic migrants from malaria-endemic regions regularly cross the border and settle temporarily in the Embera-Wounan comarca. In fact, movement of malaria across the porous eastern border with Colombia poses a major obstacle to achieving malaria elimination in Panama. The higher malaria incidence and parasite diversity observed in the neighboring country can heavily impact malaria transmission and the risk of getting infected, hindering malaria elimination efforts in Panama. 

This situation highlights the need for setting a common agenda for malaria elimination across the internationally boundary, and the development of an integrated policy between Colombia and Panama. To attain maximum benefit, malaria control and preventive activities need to be synchronized between both countries. Similar strategies have been successfully implemented for the mobile Ngäbe populations that inhabits the Ngäbe-Bugle comarca in the western side of the country, many of whom seasonally migrate across the Panama/Costa Rica border for economic reasons

In this line, some regional peculiarities have also been described in the species richness and geographic distribution of anopheline vectors in both sides of the country. For example, although *An. albimanus* and *An. punctimacula* are the most abundant and widespread vectors in both areas, other species such as *An. darlingi* have only been found in eastern endemic regions closer to the Colombia border [31,32]. Furthermore, *An. vestitipennis* and *An. neivai*, which are confirmed malaria vectors in the northern Mesoamerican countries of Belize [33], Mexico [34], and Honduras [35], have predominantly been described in western Panama, where they seem to cluster with specific types of vegetation and land-use practices [31]. Information regarding differential vector distribution is important as the circulation of the malaria parasites not only follows the spatial distribution of their anophelines vectors, but also because differential susceptibility of different mosquito species to infections by vk210 and vk240 variants have been described [36], which might be a factor contributing to the parasite genetic structure prevalent in Panama.

Socio-demographic variables and the geographical location of the areas where the local samples were collected might have also contributed to some extent to the parasite genetic structure observed in the different endemic regions of the country. The proximity of Darién and Guna Yala to the border of Colombia, where vector diversity is higher [32,37], can be a factor that has contributed to the higher transmission intensity and parasite diversity observed in this region of the country. Moreover, as we already mentioned, over the past years, the eastern area of the country has been facing a surge of extracontinental migrants that enter through the Darien Gap with the intention to reach the United States or Canada (Figure 1) [14]. In many of these migrants, *Plasmodium*-imported infections have been detected carrying parasites with a different genetic variant than the local ones [15,38]. This situation might have been adding to the gene pool of the parasites in an area, resulting in the establishment of new genotypes in this region of the country as described in South America [39]. Particularly in *P. vivax* infections, the long incubation periods, relapses, and early gametocytemia of this species of parasites can facilitate cross-fertilization and recombination events of different parasite genotypes within *Anopheles* vectors prevalent in the region, resulting in greater genetic variation [40] and increasing the chances for the appearance of genotypes more virulent or resilient to control.

Our study presented a number of important limitations that need to be stated. First, the study sample size was rather small and, therefore, our results do not necessarily represent the genetic diversity of *P. vivax* that could be seen in the country nor on the endemic regions evaluated. For this reason, we cannot rule out the presence of other genotypes in regions where they were not identified, although overall patterns of increased diversity in eastern Panama when compared with western Panama are likely robust. Second, the *Plasmodium* genes to be analyzed and the detection method to be used for genotyping should be carefully chosen by the malaria elimination programs from Mesoamerican countries. Otherwise, results between countries will not be comparable and the information will be useless for the elimination regional goal. As stated, in our study, visual detection of different genotypes in agarose gels was rather subjective. 

Our descriptive data suggest a geographical difference in distribution of *P. vivax* genotypes between the western and eastern endemic regions of the country. However, to properly address and elucidate this finding, and to determine the possible contribution of imported genotypes in the local parasite diversity, a larger number of samples representative of all endemic regions must be analyzed. Furthermore, a more robust analytical method such as analysis of molecular variance (AMOVA) is required to specifically address these questions.

A recent study using selective whole genome amplification along with SNPs selection evaluated 59 *P. vivax* samples from Panama and found a low diversity, with most of the samples belonging to a single highly related lineage that has persisted throughout endemic regions in Panama for a least a decade [38]. Nevertheless, our study revealed a higher genetic diversity among parasites from different endemic regions, particularly in eastern regions, when analyzing the *P. vivax* genes (*csp*, *msp*-1, and *msp*-3α). These three polymorphic genes have been widely used and have proven to be suitable to assess and compare genotypes of *P. vivax* infecting humans and vectors across endemic regions from different latitudes [16]. Discrepancies between both studies might be related to the use of markers that exhibit dissimilar mutational rates and that present differences in the information provided to assess diversity.

Unlike methods based on genome-wide sequencing, monitoring genetic diversity using the markers and protocols used in this study can be easily implemented through Mesoamerica and Hispaniola Island. This is because these methods are based on simple PCR reactions that can be implemented with equipment, i.e., thermocyclers, available in all national reference laboratories in the region, and sequencing of samples can be done using Sanger methods that can be processed at a low cost (10 US dollars/sample) if sent abroad, or regional cooperation could further reduce prices, using sequencers in reference laboratories that routinely sequence genetic markers of pathogens, as currently done in the ICGES [15]. Beyond significant savings in molecular genotyping, building a functional network of molecular malaria surveillance could help building a stronger regional malaria surveillance with standardized molecular protocols that clearly uncover the flow of parasites in the region, allowing for the identification of areas that serve as sources of infection and that require attention for successfully eliminating malaria from Mesoamerica. 

## 4. Materials and Methods

### 4.1. Study Site 

Panama lies at the southeastern end on Mesoamerica, joining southern North America with South America (Figure 1). Because of the construction and opening of the Panama Canal in 1914 (an artificial waterway that connects the Atlantic Ocean with the Pacific Ocean), the country has been artificially split into two sides referred as eastern and western Panama (Figure 1). During the last decades, *P. falciparum* and *P. vivax* have cohabited in malaria-endemic areas located to the east of the Panama Canal, closer to the Colombian border. Interestingly, to the west of the Panama Canal, closer to the Central American countries, only *P. vivax* transmission has been registered since the early 1960s [10]. Geographically, most malaria cases (around 90%) concentrate in the comarcas. The comarcas are semi-autonomous areas inhabited by diverse indigenous groups that occupy 22% of the national territory and together represent around 12% of the total Panamanian population [10]. *Plasmodium vivax* infections account for 90% of the total malaria cases registered in the country in the last 20 years [10]. 

### 4.2. Sample Collection and Plasmodium Diagnosis

Malaria samples and epidemiological data analyzed in this study were collected by the National Malaria Control Program (NMCP) personnel as part of the routine surveillance performed in all endemic areas. Clinical blood samples were collected by finger-pricks between 2007 and 2020 from malaria patients, including autochthonous and imported cases, the latter being inferred by the case travel history. Blood samples were used to prepare thick blood smears for routine diagnosis and spotted onto filter paper for molecular analysis. Giemsa-stained smears were examined for *Plasmodium* species, parasite density, and the presence of gametocytes following the national guidelines for malaria control of the Ministry of Health of Panama [41]. Parasitemia was classified as low, moderate, or high according to the number of parasites observed per microscopic field [42]. The following epidemiological data were also captured from every patient: geographical location, age, sex, date of sample collection, and presumed origin of infection (autochthonous vs. imported) based on the patients’ travel history.

Genomic DNA was extracted from samples with the commercial kit QIAamp DNA mini kit (Qiagen, Hilden, Germany) following the protocol for dried spot blood recommended by the manufacturer. To confirm *P. vivax* positive smear samples, we used a nested PCR that amplifies the small subunit ribosomal ribonucleic acid (ssrRNA) genes, following a slightly modified methodology from the protocol by Snounou et al. [43]. Specifically, as *P. vivax* and *P. falciparum* are the only malaria species circulating in Panama, in the second PCR reaction, only specific primers for both species were included. All amplification reactions were carried out in a total volume of 50 µL using the premixed solution PCR Nucleotide Mix (Promega, USA). Additionally, 0.4% of BSA was added to the reaction along with an extra quantity of MgCl_2_ to reach a final concentration of 2.0 mM. 

### 4.3. Plasmodium Vivax Genotyping and Csp Phylogenetic Analysis

Genetic diversity of confirmed *P. vivax* samples was assessed by genotyping the conventional polymorphic markers: *csp*, *msp*-1, and *msp-3*α. Standard nested PCR-RFLP strategies were initially applied to evaluate diversity within these three genes using primers and protocols previously described [44]. To distinguish the two types of *P. vivax* repeats in the *csp* gene (vk210 and vk247), nested PCR products were separately digested with *BstN* I (Promega, USA) following the manufacturer’s instructions [44]. To evaluate polymorphism in the interallelic variable fragment F2 of the *msp-1* gene, the nested PCR products were digested with *Alu* I (Promega, USA) following the manufacturer’s instructions [44]. For *msp*-3 α allelic diversity assessment, the second PCR products were digested with *Hha* I (Promega, USA) [45]. Digestion products were electrophoresed on 2% agarose gels containing ethidium bromide, and alleles of each gene were assigned based on size and the restriction banding patterns observed. To minimize bias, gels were analyzed independently by at least two researchers and gels were run with an appropriate molecular size marker for comparison. Haplotypes were assigned in sequential numerical orders by combining the genetic variants detected in the three loci (*csp*, *msp*-1, and *msp-3*α) analyzed in this study.

To further evaluate diversity in the *P. vivax* samples, the *csp* gene was sequenced. For this purpose, the *csp* gene was amplified as previously described [44]. The nested PCR products were electrophoresed on a 1.5% agarose gel, purified using a Qiagen DNA purification kit (Qiagen, MD, USA), and then directly sequenced in both directions with the Sanger method using an ABI Prism 3500 XL130 sequencer (Applied Biosystems, Foster City, CA, USA). Nucleotide or amino acid sequences were edited and aligned with the Sequencher 4.1.4 and Molecular Evolutionary Genetics Analysis (MEGA) 7.0 software (Pennsylvania State University, Center, PA, USA). Phylogenetic trees were constructed using MEGA 7.0 by the maximum likelihood method with 1000 bootstrap replicates. Nucleotide sequences of *csp* from this study were submitted and registered in GenBank under accession numbers MW556323-MW556428 and MW847285-MW847305.

### 4.4. Data and Statistical Analysis

The frequency of *csp*, *msp*-1, and *msp-3*α alleles was calculated as the proportion of the allele detected for each allelic family out of the total alleles detected. The frequency of polyclonal infection was calculated based on the number of samples with more than one amplified fragment in the nested PCR, or by analyzing the restriction patterns after cutting with the respective enzyme. 

A multiple correspondence analysis (MCA) was performed to analyze the pattern of association between haplotypes, built by combining genetic variants of the loci under study, and malaria epidemiological parameters. The MCA was fitted with the command mca from the library MASS [15] using the statistical software R version 3.6.1. Briefly, the MCA is a tool that allows us to study the association between categorical variables, and it is estimated by performing a singular value decomposition (SVD) on a table with counts for categories from the different studied variables that co-occur across the studied subjects [24]. Then, the original data can be projected into the two vectors associated with the largest singular values from the SVD. The resulting values are “coordinates”, including centroids for all levels from the different categorical variables considered in the analysis. The centroids are plotted in two dimensions, thus allowing for the evaluation of associations between categories from different variables [15,46]. Associations are stronger as categories from different variables are together but farther apart from the origin (coordinates 0, 0 in the 2D plot), which is the geometric point where random associations are expected to appear [24]. Variables considered in this analysis were as follows: age (grouped in three categories, children: 1 to 5 years, infants: 6 to 15 years, and adults: 16 or more), gender (male or female), parasite load (without parasites, low, medium, or high), gametocyte presence, date (year), season (rainy or dry), and presumed origin of infection (autochthonous, including the malaria case origin province/comarca, or imported, based solely on the patients travel history).

### 4.5. Ethical Statement

The retrospective molecular analysis of the samples and the protocols used for molecular surveillance were approved by the Departamento de Control de Vectores of the Ministry of Health (No. 147/DCV/DGSP and No. 374/DCV/ICG) and by the Comité de Bioética de la Investigación del Instituto Conmemorativo Gorgas de Estudios de la Salud (No. 468/CNBI/ICGES/06 and No. 413/CNBI/ICGES/12). The search of suspected cases, diagnostic, treatment, and documentation procedures of each malaria case (detected via active and passive surveillance) were conducted by the Departamento de Control de Vectores as part of the routine surveillance system for malaria control. Epidemiological information was also obtained from the Departamento de Control de Vectores from the Ministry of Health databases. The confidentiality of the study subjects with malaria was protected and individual data were not shared. 

## Figures and Tables

**Table 1:** Allelic frequencies of *csp*, *msp*-1, and *msp*-3α genes in *Plasmodium vivax* isolates from malaria-endemic regions in Panama and in imported cases based on patients’ travel history.

	*Csp* Vk210	*Csp* Vk247	*Msp-1* Genotype G1	*Msp-1* Genotype G2	*Msp-1* Genotype G3	*Msp-1* Genotype G4	*Msp-3α* Genotype G1	*Msp-3α* Genotype G2	*Msp-3α* Genotype G3
Bocas del Toro	12 (5.8%)	0	15 (6.4%)	4 (1.7%)	0	0	13 (5.5%)	0	0
Veraguas	25 (12%)	0	25 (10.7%)	0	0	0	25 (10.6%)	0	0
Panamá Metro	17 (8.2%)	0	15 (6.4%)	1 (0.4%)	0	0	16 (6.8%)	0	0
Panamá Este	40 (19.2%)	0	42 (17.9%)	5 (2.1%)	0	0	43 (18.2%)	2 (1%)	0
Guna Yala	29 (13.9%)	5 (2.4%)	22 (9.4%)	3 (1.3%)	0	0	15 (6.3%)	11 (4.7%)	0
Darién	35 (16.9%)	29 (13.9%)	48 (20.5%)	23 (9.8%)	0	0	53 (22.4%)	25 (10.6%)	1 (0.4%)
Imported	12 (5.8%)	4 (1.9%)	10 (4.3%)	18 (7.7%)	1 (0.4%)	2 (1%)	26 (11%)	5 (2.1%)	1 (0.4%)
Total	170	38	177	54	1	2	191	43	2

**Table 2:** Haplotype diversity and distribution inferred by combining the alleles detected in the three loci in *Plasmodium vivax* indigenous and imported cases based on travel history.

	*Csp* Genotype	*Msp*-1 Genotype	*Msp*-3α Genotype	Bocas del Toro	Veraguas	Panamá Metro	Panamá Este	Guna Yala	Darién	Imported	Total (%)
**Haplotype 1**	vk210	G2	G1			1			4		5 (3.1)
**Haplotype 2**	vk247	G2	G2					1	1		2 (1.2)
**Haplotype 3**	vk247	G2	G1						1		1 (0.6)
**Haplotype 4**	vk247	G1	G2						6	1	7 (4.3)
**Haplotype 5**	vk247	G1	G1						16		16 (9.8)
**Haplotype 6**	vK210	G1	G2						7		7 (4.3)
**Haplotype 7**	vK210	G2	G1					1	9	6	16 (9.8)
**Haplotype 8**	vk247	G3	G3							1	1 (0.6)
**Haplotype 9**	vk247	G4	G1							1	1 (0.6)
**Haplotype 10**	vk210	G1	G1	12	25	14	38	8	8	2	107 (65.6)
**Total**				12	25	15	38	10	52	11	163

**Table 3:** Distribution and frequency of *csp* vk210 subtypes according to the origin of the isolate.

	Nonapeptide Variant					Isolate Origin				
Vk210 Subtype	A	B	C	D	E	WP	EP	Imp	Total	%
Allele 1	10	9		1			3	2	5	7.8
Allele 2	10	7			2			1	1	1.6
Allele 3	11	7		1		7	27	8	42	65.6
Allele 4	16	3		1			1	1	2	3.1
Allele 5	10	7			1			1	1	1.6
Allele 6	6	10		1			1		1	1.7
Allele 7	12	5		1				1	1	1.6
Allele 8	18			1				1	1	1.6
Allele 9	12	6		1		1	1		2	3.1
Allele 10	16							2	2	3.1
Allele 11	10	7				2			2	3.1
Allele 12	13	4				2			2	3.1
Allele 13	15		1	1				1	1	1.6
Allele 14	14		2	2			1		1	1.6
Total						12	34	18	64	100

WP: West Panama (includes samples from Bocas del Toro and Veraguas); EP: East Panama includes samples from (Panamá Este, Guna Yala, and Darién); Imp: Imported (includes isolates collected from suspected imported cases based on their travel history). Nonapeptide variants: A: GDRADGQPA; B: GDRAAGQPA; C: GNRADGQPA; D: GNGAGGQAA; E: GDRAAGQAA. The presence of a particular variant within each allele is represented by boxes with gray background and the number of repeats is shown inside each of these boxes.

## Data Availability

All data underlying results from this study are provided as part of the article in tables and figures. DNA sequences were deposited in GenBank as described in the methodology.

## Supplementary Materials

The following are available online at https://www.mdpi.com/article/10.3390/pathogens10080989/s1, Figure S1: Genotyping of *Plasmodium vivax* infections by PCR-RFLP: (a) merozoite surface proteins 1 (*msp*-1), (b) merozoite surface proteins 3 (*msp*-3α), and (c) circumsporozoite protein (*csp*). Figure S2: PCR-RFLP profiles of *msp*-1 gene amplified from *P. vivax* study samples digested with the Alu I enzyme. Figure S3: PCR-RFLP profiles of *msp*-3α gene amplified from *P. vivax* study samples digested with *Hha* I enzyme.
