# Supplementary material for: Plasmodium vivax Genetic Diversity in Panama: Challenges for Malaria Elimination in Mesoamerica
Source: Pathogens. 2021 Aug 5;10(8):989. doi: 10.3390/pathogens10080989 (PMC8401434; doi:10.3390/pathogens10080989)
Supplement: Supplementary file 1 [file pathogens-10-00989-s001.zip › Figure S1 revised.pdf]

a.

1 2 3 4 5 6 7 8

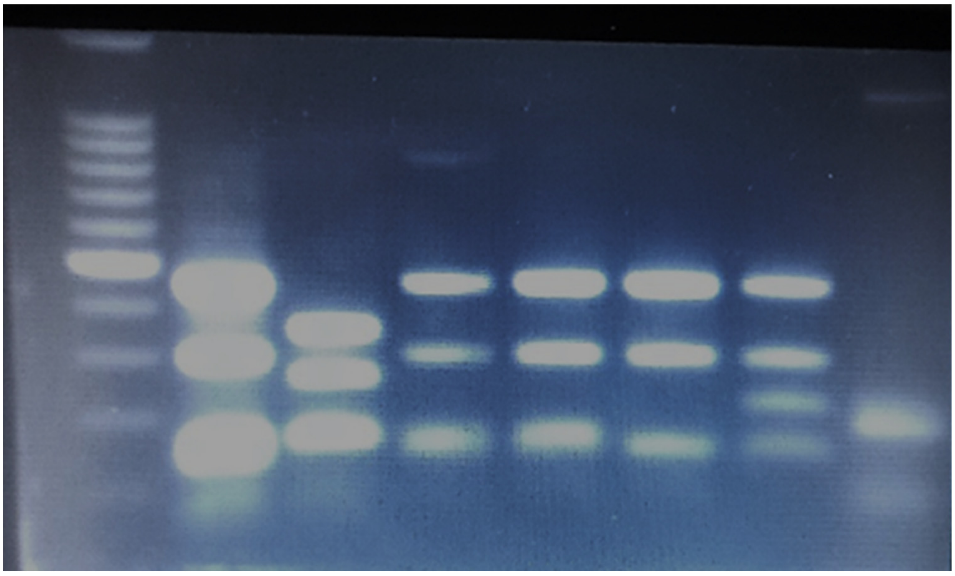

b.

1 2 3 4 5

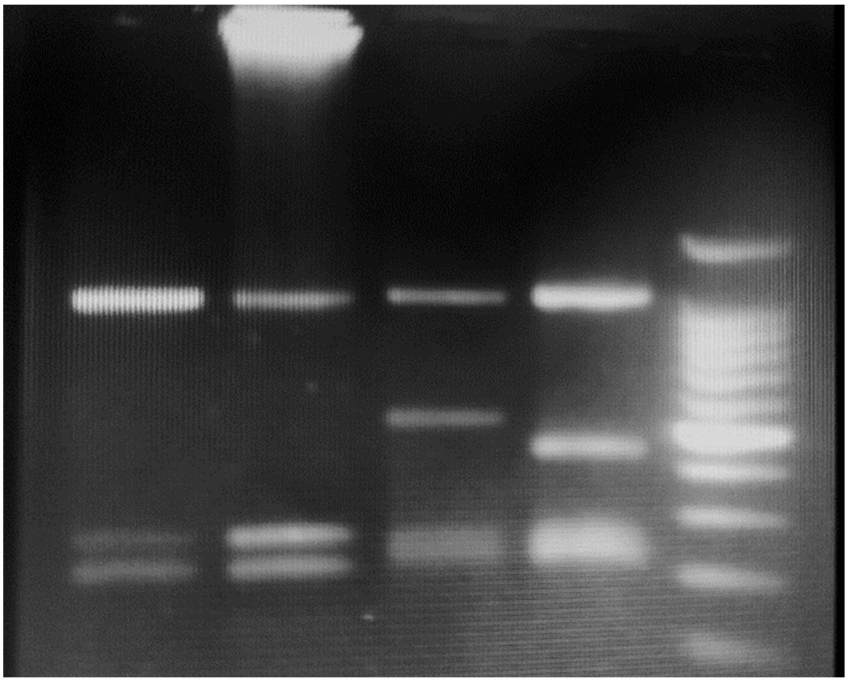

c.

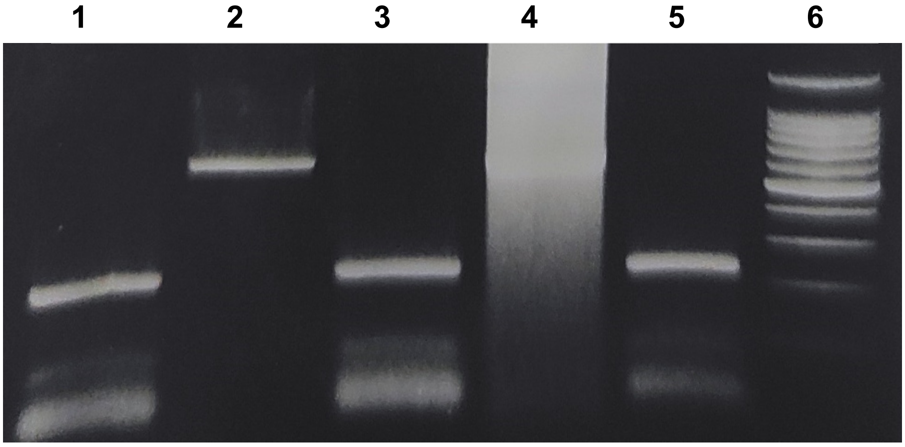

a. Representative agarose gel for *Plasmodium vivax msp*-1 gene products digested with *Alu* I. Lane 1: Molecular weight marker 100-bp ladder; Lanes 2, 4, 5 and 6: Genotype I; Lane 3: Genotype II; Lane 7: Genotype III; Lane 8: Genotype IV.

b. Representative agarose gel for *Plasmodium vivax msp*-3α gene products digested with *Hha* I. Lanes 1 and 2: Genotype II; Lane 3: Genotype 1; Lane 4: Genotype III; Lane 5: Molecular weight marker 100-bp ladder.

c. Representative agarose gel for *Plasmodium vivax csp* gene products digested with *BstN* I. Lanes: 1, 3, and 5: vk210 variant; Lanes 2 and 4: Undigested amplification products; Lane 5: Molecular weight marker 100-bp ladder.
