# Supplementary material for: Plasmodium vivax Genetic Diversity in Panama: Challenges for Malaria Elimination in Mesoamerica
Source: Pathogens. 2021 Aug 5;10(8):989. doi: 10.3390/pathogens10080989 (PMC8401434; doi:10.3390/pathogens10080989)

Figure S2. PCR-RFLP profiles of *msp-1* gene amplified from *P. vivax* study samples digested with *Alu* I enzyme.

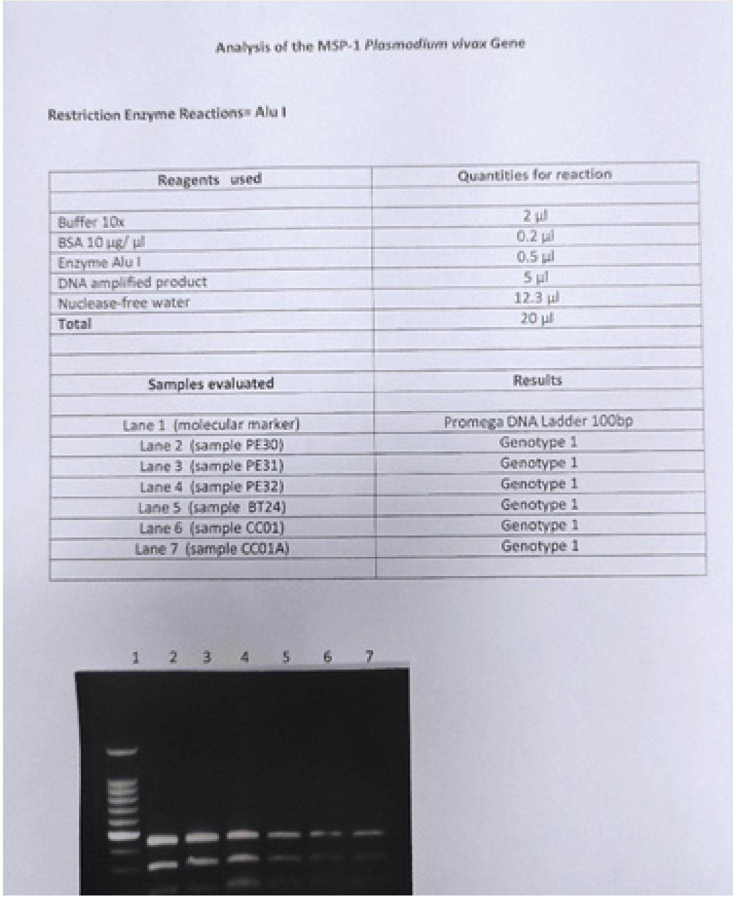

Analysis of the MSP-1 *Plasmodium vivax* Gene

Restriction Enzyme Reactions= Alu I

Reagents used	Quantities for reaction
Buffer 10x	2 µl
BSA 10 µg/ µl	0.2 µl
Enzyme Alu I	0.5 µl
DNA amplified product	5 µl
Nuclease-free water	12.3 µl
**Total**	20 µl
**Samples evaluated**	**Results**
Lane 1 (molecular marker)	Promega DNA Ladder 100bp
Lane 2 (sample PE30)	Genotype 1
Lane 3 (sample PE31)	Genotype 1
Lane 4 (sample PE32)	Genotype 1
Lane 5 (sample BT24)	Genotype 1
Lane 6 (sample CC01)	Genotype 1
Lane 7 (sample CC01A)	Genotype 1

Analysis of the MSP-1 *Plasmodium vivax* Gene

Restriction Enzyme Reactions= Alu I

**Reagents used**	**Quantities for reaction**
Buffer 10x	2 µl
BSA 10 µg/ µl	0.2 µl
EnzymeAlu I	0.5 µl
DNA amplified product	5 µl
Nuclease-free water	12.3 µl
**Total**	20 µl
**Samples evaluated**	**Results**
Lane 1 (molecular marker)	Promega DNA Ladder 100bp
Lane 2 (sample DA28)	Genotype I
Lane 3 (sample DA40)	Genotype 2
Lane 3 (sample DA30)	Genotype I
Lane 4 (sample DA31)	Genotype I
Lane 5 (sample DA32)	Genotype I

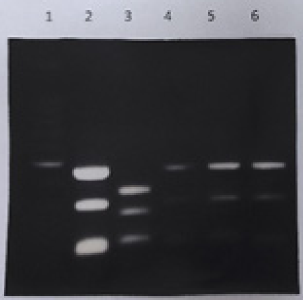

Analysis of the MSP-1 *Plasmodium vivax* Gene

Restriction Enzyme Reactions= Alu I

**Reagents used**	**Quantities for reaction**
Buffer 10x	2 µl
BSA 10 µg/ µl	0.2 µl
EnzymeAlu I	0.5 µl
DNA amplified product	5 µl
Nuclease-free water	12.3 µl
**Total**	20 µl
**Samples evaluated**	**Results**
Lane 1 (molecular marker)	Promega DNA Ladder 100bp
Lane 2 (sample DA44)	Genotype 2
Lane 3 (sample PO01)	Genotype 3
Lane 3 (sample PE32)	Genotype I

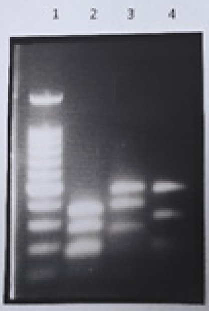

Analysis of the MSP-1 *Plasmodium vivax* Gene

Restriction Enzyme Reactions= Alu I

Reagents used	Quantities for reaction
Buffer 10x	2 µl
BSA 10 µg/ µl	0.2 µl
Enzyme Alu I	0.5 µl
DNA amplified product	5 µl
Nuclease-free water	12.3 µl
**Total**	20 µl
**Samples evaluated**	Results
Lane 1 (molecular marker)	Promega DNA Ladder 100bp
Lane 2 (sample KY145)	Genotype 1
Lane 3 (sample KY147)	Genotype 1
Lane 4 (simple KY160)	Genotype 1
Lane 5 (sample KY161)	Genotype 1
Lane 6 (sample KY163)	Genotype 1

1 2 3 4 5 6

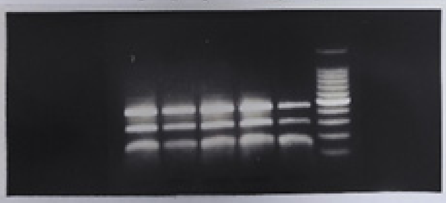

Analysis of the MSP-1 *Plasmodium vivax* Gene

Restriction Enzyme Reactions= Alu I

**Reagents used**	**Quantities for reaction**
Buffer 10x	2 µl
BSA 10 µg/ µl	0.2 µl
Enzyme Alu I	0.5 µl
DNA amplified product	5 µl
Nuclease-free water	12.3 µl
**Total**	20 µl
**Samples evaluated**	Results
Lane 1 (molecular marker)	Promega DNA Ladder 100bp
Lane 2 (sample PE60)	Genotype 2
Lane 3 (sample PE58)	Genotype 2
Lane 4 (sample PE65)	Genotype 1
Lane 5 (sample BT16)	Genotype 1
Lane 6 (simple DA25)	Genotype 1

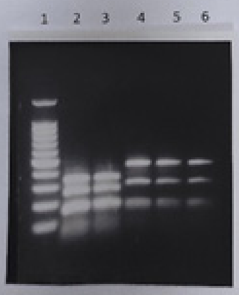

Analysis of the MSP-1 *Plasmodium vivax* Gene

Restriction Enzyme Reactions= Alu I

Reagents used	Quantities for reaction
Buffer 10x	2 µl
BSA 10 µg/ µl	0.2 µl
Enzyme Alu I	0.5 µl
DNA amplified product	5 µl
Nuclease-free water	12.3 µl
**Total**	20 µl
**Samples evaluated**	Results
Lane 1 (molecular marker)	Promega DNA Ladder 100bp
Lane 2 (sample PE34)	Genotype 1
Lane 3 (sample PE35)	Genotype 1
Lane 4 (sample PE36)	Genotype 1
Lane 5 (sample BT08)	Genotype 1
Lane 6 (sample BT12)	Genotype 1
Lane 7 (sample BT13)	Genotype 1
Lane 8 (sample DA06)	Genotype 1
Lane 9 (sample DA08)	Genotype 1
Lane 10 (simple DA09)	Genotype 1
Lane 11 (sample DA14)	Genotype 1
Lane 12 undigested secondary PCR product	undigested product

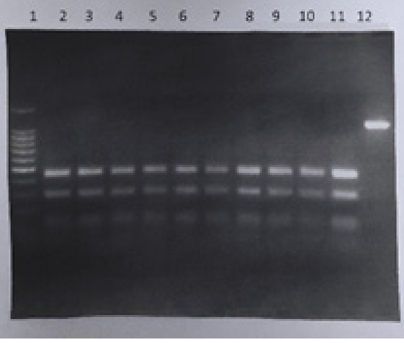

Analysis of the MSP-1 *Plasmodium vivax* Gene

Restriction Enzyme Reactions= Alu I

Reagents used	Quantities for reaction
Buffer 10x	2 µl
BSA 10 µg/ µl	0.2 µl
Enzyme Alu I	0.5 µl
DNA amplified product	5 µl
Nuclease-free water	12.3 µl
**Total**	20 µl
**Samples evaluated**	Results
Lane 1 (sample KY163)	repeat sample
Lane 2 (sample KY153)	Genotype 1
Lane 3 (sample KY157)	Genotype 1
Lane 4 (sample KY160)	repeat sample
Lane 5 undigested secondary PCR product	undigested product
Lane 6 (molecular marker)	Promega DNA Ladder 100bp

1 2 3 4 5 6

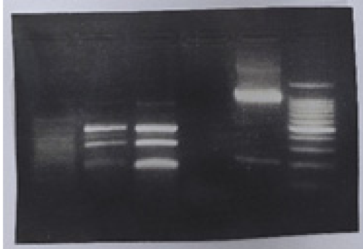

Analysis of the MSP-1 Plasmodium vivax Gene

Restriction Enzyme Reactions= Alu I

Reagents used	Quantities for reaction
Buffer 10x	2 µl
BSA 10 µg/ µl	0.2 µl
Enzyme Alu I	0.5 µl
DNA amplified product	5 µl
Nuclease-free water	12.3 µl
**Total**	20 µl
**Samples evaluated**	**Results**
Lane 1 (molecular marker)	Promega DNA Ladder 100bp
Lane 2 (sample DOBA17)	Genotype 1
Lane 3 (sample PO05)	Genotype 2
Lane 4 (sample VE24)	Genotype 1
Lane 5 (sample VE23)	Genotype 1
Lane 6 (sample VE21)	Genotype 1
Lane 7 (sample PO01)	Genotype 3
Lane 8 (sample PO06)	Genotype 4

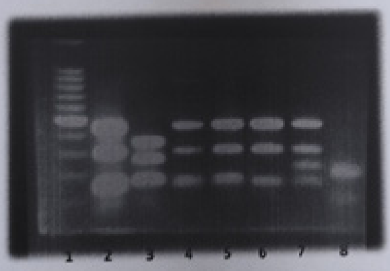

Analysis of the MSP-1 *Plasmodium vivax* Gene

Restriction Enzyme Reactions= Alu I

**Reagents used**	**Quantities for reaction**
Buffer 10x	2 µl
BSA 10 µg/ µl	0.2 µl
Enzyme Alu I	0.5 µl
DNA amplified product	5 µl
Nuclease-free water	12.3 µl
**Total**	20 µl
**Samples evaluated**	**Results**
Lane 1 (molecular marker)	Promega DNA Ladder 100bp
Lane 2 (sample PO56)	Genotype 2
Lane 3 (sample PO31)	Genotype 2
Lane 4 (sample PE39)	Genotype 1
Lane 5 (sample PO22)	Genotype 1
Lane 6 (sample PO29)	Genotype 1
Lane 7 (sample PO48)	Genotype 1
Lane 8 (sample PO01)	Genotype 3
Lane 9 (sample PO01)	Genotype 3
Lane 10 (sample PO07)	Genotype 1

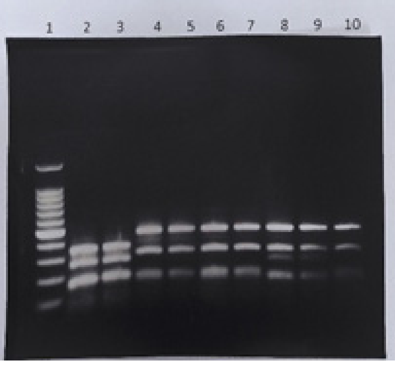

Analysis of the MSP-1 *Plasmodium vivax* Gene

Restriction Enzyme Reactions= Alu I

**Reagents used**	**Quantities for reaction**
Buffer 10x	2 µl
BSA 10 µg/ µl	0.2 µl
Enzyme Alu I	0.5 µl
DNA amplified product	5 µl
Nuclease-free water	12.3 µl
**Total**	20 µl
**Samples evaluated**	**Results**
Lane 1 (molecular marker)	Promega DNA Ladder 100bp
Lane 2 (sample DA67)	Genotype 2
Lane 3 (sample DA68)	Genotype 2
Lane 4 (sample PD48)	Genotype 1

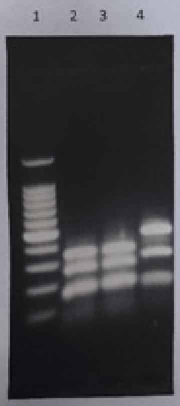

Analysis of the MSP-1 *Plasmodium vivax* Gene

Restriction Enzyme Reactions= Alu I

Reagents used	Quantities for reaction
Buffer 10x	2 µl
BSA 10 µg/ µl	0.2 µl
Enzyme Alu I	0.5 µl
DNA amplified product	5 µl
Nuclease-free water	12.3 µl
**Total**	20 µl
**Samples evaluated**	Results
Lane 1 (sample VE007)	Genotype 1
Lane 2 (sample VE008)	Genotype 1
Lane 3 (sample VE009)	Genotype 1
Lane 4 (sample VE010)	Genotype 1
Lane 5 (sample VE011)	Genotype 1
Lane 6 (molecular marker)	Promega DNA Ladder 100bp

1 2 3 4 5 6

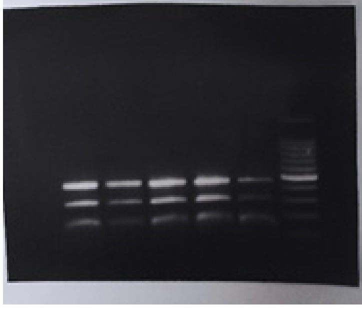

Analysis of the MSP-1 Plasmodium vivax Gene

Restriction Enzyme Reactions= Alu I

**Reagents used**	**Quantities for reaction**
Buffer 10x	2 µl
BSA 10 µg/ µl	0.2 µl
Enzyme Alu I	0.5 µl
DNA amplified product	5 µl
Nuclease-free water	12.3 µl
**Total**	20 µl
**Samples evaluated**	**Results**
Lane 1 (molecular marker)	Promega DNA Ladder 100bp
Lane 2 (sample PE30)	Genotype 1
Lane 3 (sample PE31)	Genotype 2
Lane 4 (sample PE32)	Genotype 1
Lane 5 (sample BT24)	Genotype 1
Lane 6 (sample CC01)	Genotype 1
Lane 7 (sample PO01)	Genotype 3
Lane 8 (sample CC01 A)	Genotype 1

1 2 3 4 5 6 7 8

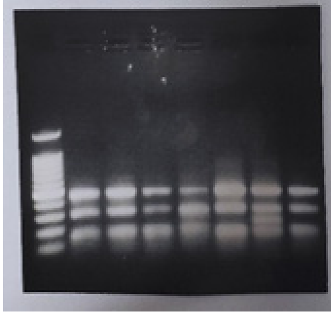

Analysis of the MSP-1 *Plasmodium vivax* Gene

Restriction Enzyme Reactions= Alu I

Reagents used	Quantities for reaction
Buffer 10x	2 µl
BSA 10 µg/ µl	0.2 µl
Enzyme Alu I	0.5 µl
DNA amplified product	5 µl
Nuclease-free water	12.3 µl
**Total**	20 µl
**Samples evaluated**	Results
Lane 1 (molecular marker)	Promega DNA Ladder 100bp
Lane 2 (sample PO62)	Genotype 1
Lane 3 (sample PO59)	Genotype 1
Lane 4 (molecular marker)	Promega DNA Ladder 100bp
Lane 5 undigested secondary PCR product	undigested product
Lane 6 undigested secondary PCR product	undigested product

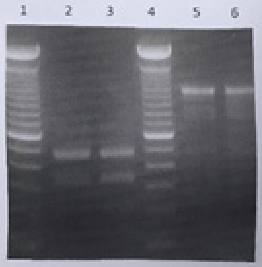

Analysis of the MSP-1 *Plasmodium vivax* Gene

Restriction Enzyme Reactions= Alu I

**Reagents used**	**Quantities for reaction**
Buffer 10x	2 µl
BSA 10 µg/ µl	0.2 µl
Enzyme Alu I	0.5 µl
DNA amplified product	5 µl
Nuclease-free water	12.3 µl
**Total**	20 µl
**Samples evaluated**	**Results**
Lane 1 (sample KY173)	Genotype 1
Lane 2 (sample KY168)	Genotype 1
Lane 3 (sample KY169)	Genotype 1
Lane 4 (molecular marker)	Promega DNA Ladder 100bp

1 2 3 4

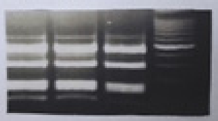

Analysis of the MSP-1 *Plasmodium vivax* Gene

Restriction Enzyme Reactions= Alu I

Reagents used	Quantities for reaction
Buffer 10x	2 µl
BSA 10 µg/ µl	0.2 µl
Enzyme Alu I	0.5 µl
DNA amplified product	5 µl
Nuclease-free water	12.3 µl
**Total**	20 µl
**Samples evaluated**	Results
Lane 1 (molecular marker)	Promega DNA Ladder 100bp
Lane 2 (sample KY130)	Genotype 1
Lane 3 (sample KY131)	Genotype 1
Lane 4 (sample KY136)	Genotype 1
Lane 5 (sample DA11)	Genotype 1
Lane 6 (sample DA12)	Genotype 1
Lane 7 (sample DA13)	Genotype 1
Lane 8 (sample DOBA07)	Genotype 1
Lane 9 (sample DOBA09)	Genotype 1
Lane 10 (simple DOBA15)	Genotype 1
Lane 11 (sample PO50)	Genotype 1
Lane 12 (sample PO54)	Genotype 1

1 2 3 4 5 6 7 8 9 10 11 12

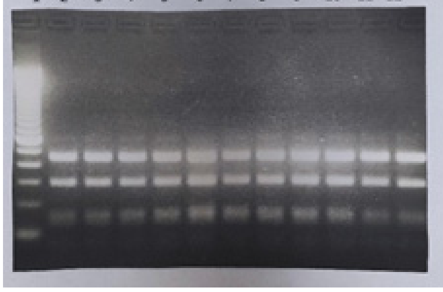

Analysis of the MSP-1 *Plasmodium vivax* Gene

Restriction Enzyme Reactions= Alu I

Reagents used	Quantities for reaction
Buffer 10x	2 µl
BSA 10 µg/ µl	0.2 µl
EnzymeAlu I	0.5 µl
DNA amplified product	5 µl
Nuclease-free water	12.3 µl
**Total**	20 µl
**Samples evaluated**	Results
Lane 1 (molecular marker)	Promega DNA Ladder 100bp
Lane 2 (sample DA17)	Genotype I
Lane 3 (sample DA18)	Genotype I
Lane 4 (sample DA19)	Genotype I
Lane 5 (sample DA22)	Genotype I
Lane 6 (sample DA23)	Genotype I
Lane 7 (sample DA24)	Genotype I
Lane 8 undigested secondary PCR product	undigested product

1 2 3 4 5 6 7 8

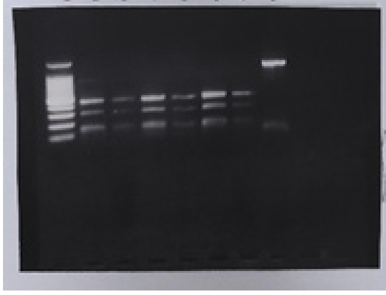

Analysis of the MSP-1 *Plasmodium vivax* Gene

Restriction Enzyme Reactions= Alu I

**Reagents used**	**Quantities for reaction**
Buffer 10x	2 µl
BSA 10 µg/ µl	0.2 µl
Enzyme Alu I	0.5 µl
DNA amplified product	5 µl
Nuclease-free water	12.3 µl
**Total**	20 µl
**Samples evaluated**	**Results**
Lane 1 (sample DA71)	Genotype 1
Lane 2 (sample DA73)	Genotype 1
Lane 3 undigested secondary PCR product	undigested product
Lane 4 (molecular marker)	Promega DNA Ladder 100bp

1 2 3 4

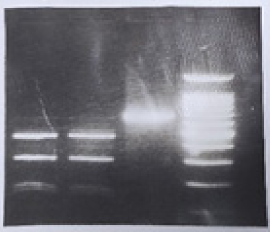

Analysis of the MSP-1 *Plasmodium vivax* Gene

Restriction Enzyme Reactions= Alu I

Reagents used	Quantities for reaction
Buffer 10x	2 µl
BSA 10 µg/ µl	0.2 µl
Enzyme Alu I	0.5 µl
DNA amplified product	5 µl
Nuclease-free water	12.3 µl
**Total**	20 µl
**Samples evaluated**	Results
Lane 1 (sample DA52)	Genotype 1
Lane 2 (simple DA53)	Genotype 1
Lane 3 (sample DA54)	Genotype 1
Lane 4 (sample DA56)	Genotype 1
Lane 5 (sample DOBA1)	Genotype 1
Lane 6 (molecular marker)	Promega DNA Ladder 100bp

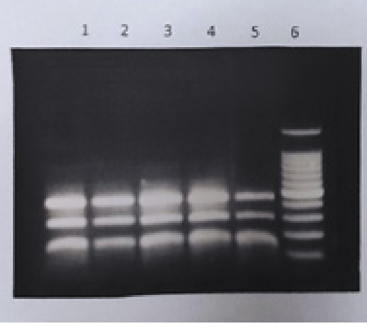

Analysis of the MSP-1 *Plasmodium vivax* Gene

Restriction Enzyme Reactions= Alu I

**Reagents used**	**Quantities for reaction**
Buffer 10x	2 µl
BSA 10 µg/ µl	0.2 µl
EnzymeAlu I	0.5 µl
DNA amplified product	5 µl
Nuclease-free water	12.3 µl
**Total**	20 µl
**Samples evaluated**	Results
Lane 1 (molecular marker)	Promega DNA Ladder 100bp
Lane 2 (sample DA39)	Genotype I
Lane 3 (sample DA42)	Genotype I
Lane 4 (sample DA43)	Genotype I
Lane 5 (sample DA45)	Genotype I
Lane 6 (sample DA47)	Genotype I
Lane 7 (sample DA48)	Genotype I
Lane 8 undigested secondary PCR product	undigested product

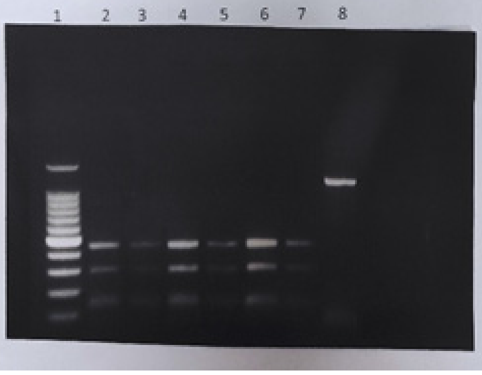

Analysis of the MSP-1 *Plasmodium vivax* Gene

Restriction Enzyme Reactions= Alu I

Reagents used	Quantities for reaction
Buffer 10x	2 µl
BSA 10 µg/ µl	0.2 µl
EnzymeAlu I	0.5 µl
DNA amplified product	5 µl
Nuclease-free water	12.3 µl
**Total**	20 µl
Samples evaluated	Results
Lane 1 (molecular marker)	Promega DNA Ladder 100bp
Lane 2 (sample PM05)	Genotype I
Lane 3 (sample PM06)	Genotype I
Lane 4 (sample PM10)	Genotype I
Lane 5 (sample PM14)	Genotype I
Lane 6 (sample PM11)	Genotype I
Lane 7 (sample PM12)	Genotype I
Lane 8 undigested secondary PCR product	undigested product

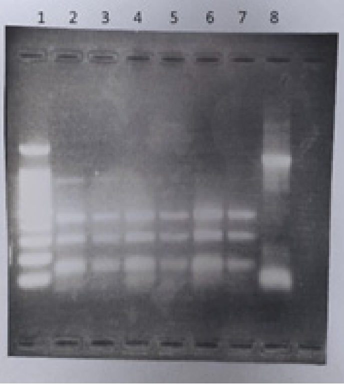

Analysis of the MSP-1 *Plasmodium vivax* Gene

Restriction Enzyme Reactions= Alu I

Reagents used	Quantities for reaction
Buffer 10x	2 µl
BSA 10 µg/ µl	0.2 µl
EnzymeAlu I	0.5 µl
DNA amplified product	5 µl
Nuclease-free water	12.3 µl
**Total**	20 µl
**Samples evaluated**	Results
Lane 1 (sample DA60)	Genotype I
Lane 2 (sample DA64)	repeat sample
Lane 3 (sample DA64)	Genotype I
Lane 4 (sample DA66)	Genotype I
Lane 5 (sample DA74)	Genotype I
Lane 6 (molecular marker)	Promega DNA Ladder 100bp

1 2 3 4 5 6

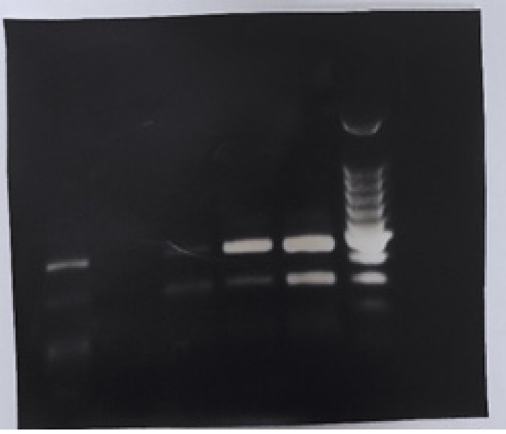

Analysis of the MSP-1 Plasmodium vivax Gene

Restriction Enzyme Reactions= Alu I

**Reagents used**	Quantities for reaction
Buffer 10x	2 µl
BSA 10 µg/ µl	0.2 µl
Enzyme Alu I	0.5 µl
DNA amplified product	5 µl
Nuclease-free water	12.3 µl
**Total**	20 µl
**Samples evaluated**	Results
Lane 1 (sample KY171)	Genotype 1
Lane 2 (sample KY172)	Genotype 1
Lane 3 (sample KY173)	Genotype 1
Lane 4 (sample KY168)	Genotype 1
Lane 5 (sample KY169)	Genotype 1
Lane 6 (molecular marker)	Promega DNA Ladder 100bp

1 2 3 4 5 6

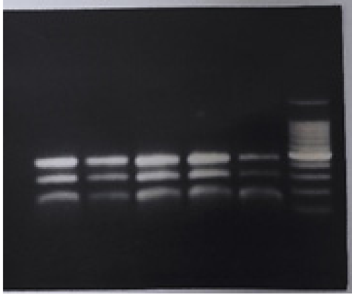

Analysis of the MSP-1 *Plasmodium vivax* Gene

Restriction Enzyme Reactions= Alu I

Reagents used	Quantities for reaction
Buffer 10x	2 µl
BSA 10 µg/ µl	0.2 µl
EnzymeAlu I	0.5 µl
DNA amplified product	5 µl
Nuclease-free water	12.3 µl
**Total**	20 µl
**Samples evaluated**	Results
Lane 1 (molecular marker)	Promega DNA Ladder 100bp
Lane 2 (sample KY139)	Genotype I
Lane 3 (sample KY140)	Genotype I
Lane 4 (sample KY141)	Genotype I
Lane 5 (sample KY142)	Genotype I
Lane 6 (sample KY143)	Genotype I
Lane 7 (sample KY144)	Genotype I
Lane 8 undigested secondary PCR product	undigested product

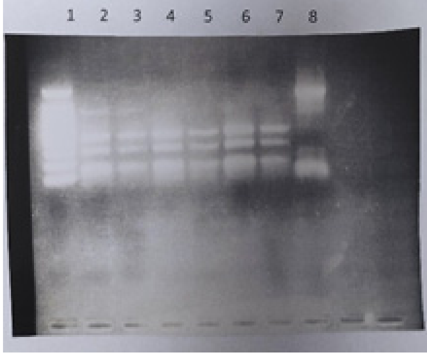

Analysis of the MSP-1 *Plasmodium vivax* Gene

Restriction Enzyme Reactions= Alu I

**Reagents used**	Quantities for reaction
Buffer 10x	2 µl
BSA 10 µg/ µl	0.2 µl
Enzyme Alu I	0.5 µl
DNA amplified product	5 µl
Nuclease-free water	12.3 µl
**Total**	20 µl
**Samples evaluated**	Results
Lane 1 (sample VE012)	Genotype 1
Lane 2 (sample VE018)	Genotype 1
Lane 3 (sample VE019)	Genotype 1
Lane 4 (sample VE020)	Genotype 1
Lane 5 (sample VE021)	Genotype 1
Lane 6 (molecular marker)	Promega DNA Ladder 100bp

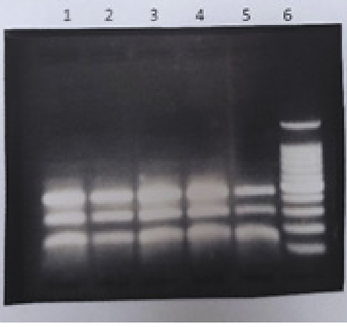

Analysis of the MSP-1 *Plasmodium vivax* Gene

Restriction Enzyme Reactions= Alu I

Reagents used	Quantities for reaction
Buffer 10x	2 µl
BSA 10 µg/ µl	0.2 µl
Enzyme Alu I	0.5 µl
DNA amplified product	5 µl
Nuclease-free water	12.3 µl
**Total**	20 µl
**Samples evaluated**	**Results**
Lane 1 (molecular marker)	Promega DNA Ladder 100bp
Lane 2 (simple BT05)	Genotype 1
Lane 3 (sample VE01)	Genotype 1
Lane 4 (sample VE02)	Genotype 1
Lane 5 (sample VE03)	Genotype 1
Lane 6 (sample PM08)	Genotype 1
Lane 7 (sample PM03)	Genotype 1
Lane 8 (sample PM04)	Genotype 1
Lane 9 (sample PE05)	Genotype 1

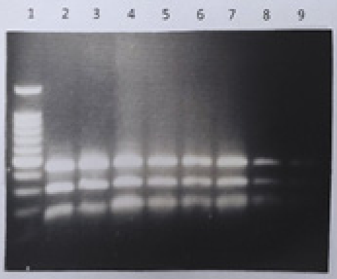

Analysis of the MSP-1 *Plasmodium vivax* Gene

Restriction Enzyme Reactions= Alu I

Reagents used	Quantities for reaction
Buffer 10x	2 µl
BSA 10 µg/ µl	0.2 µl
Enzyme Alu I	0.5 µl
DNA amplified product	5 µl
Nuclease-free water	12.3 µl
**Total**	20 µl
**Samples evaluated**	Results
Lane 1 (molecular marker)	Promega DNA Ladder 100bp
Lane 2 (sample PE50)	Genotype 2
Lane 2 (sample PM20)	Genotype 2
Lane 2 (sample PE06)	Genotype 1
Lane 3 (sample PE07)	Genotype 1
Lane 4 (sample BT01)	Genotype 1
Lane 5 (sample BT04)	Genotype 1

1 2 3 4 5 6 7

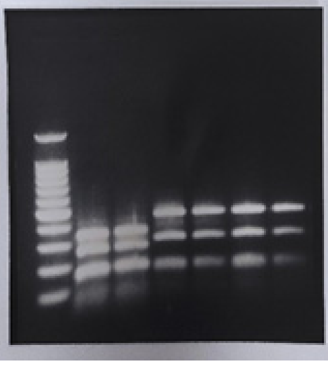

Analysis of the MSP-1 *Plasmodium vivax* Gene

Restriction Enzyme Reactions= Alu I

Reagents used	Quantities for reaction
Buffer 10x	2 µl
BSA 10 µg/ µl	0.2 µl
Enzyme Alu I	0.5 µl
DNA amplified product	5 µl
Nuclease-free water	12.3 µl
**Total**	20 µl
**Samples evaluated**	**Results**
Lane 1 (sample PE013)	Genotype 1
Lane 2 (simple PE014)	Genotype 1
Lane 3 (sample PE015)	Genotype 1
Lane 4 (sample PE016)	Genotype 1
Lane 5 (sample PE017)	Genotype 1
Lane 6 (molecular marker)	Promega DNA Ladder 100bp

1 2 3 4 5 6

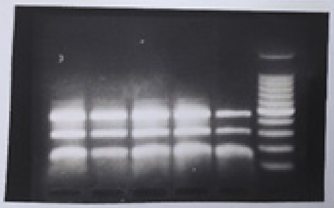

Analysis of the MSP-1 *Plasmodium vivax* Gene

Restriction Enzyme Reactions= Alu I

**Reagents used**	**Quantities for reaction**
Buffer 10x	2 µl
BSA 10 µg/ µl	0.2 µl
Enzyme Alu I	0.5 µl
DNA amplified product	5 µl
Nuclease-free water	12.3 µl
**Total**	20 µl
**Samples evaluated**	Results
Lane 1 (molecular marker)	Promega DNA Ladder 100bp
Lane 2 (sample PM19)	Genotype 1
Lane 3 (sample PM18)	Genotype 1
Lane 4 (sample PM07)	Genotype 1
Lane 5 (sample PM09)	Genotype 1
Lane 6 (sample PM13)	Genotype 1

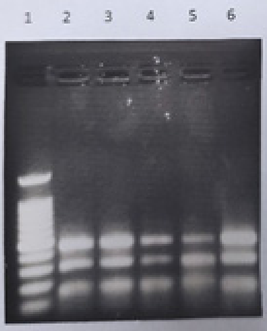

Analysis of the MSP-1 *Plasmodium vivax* Gene

Restriction Enzyme Reactions= Alu I

Reagents used	Quantities for reaction
Buffer 10x	2 µl
BSA 10 µg/ µl	0.2 µl
Enzyme Alu I	0.5 µl
DNA amplified product	5 µl
Nuclease-free water	12.3 µl
**Total**	20 µl
**Samples evaluated**	**Results**
Lane 1 (sample PE037)	Genotype 1
Lane 2 (simple PE38)	Genotype 1
Lane 3 (sample PE39)	Genotype 1
Lane 4 (sample PE40)	Genotype 1
Lane 5 (sample PE41)	Genotype 1
Lane 6 (molecular marker)	Promega DNA Ladder 100bp

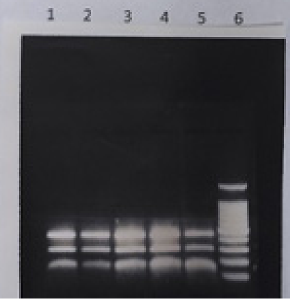

Analysis of the MSP-1 *Plasmodium vivax* Gene

Restriction Enzyme Reactions= Alu I

Reagents used	Quantities for reaction
Buffer 10x	2 µl
BSA 10 µg/ µl	0.2 µl
Enzyme Alu I	0.5 µl
DNA amplified product	5 µl
Nuclease-free water	12.3 µl
**Total**	20 µl
**Samples evaluated**	Results
Lane 1 (sample PE018)	Genotype 1
Lane 2 (simple PE025)	Genotype 1
Lane 3 (sample PE026)	Genotype 1
Lane 4 (sample PE027)	Genotype 1
Lane 5 (sample PE028)	Genotype 1
Lane 6 (molecular marker)	Promega DNA Ladder 100bp

1 2 3 4 5 6

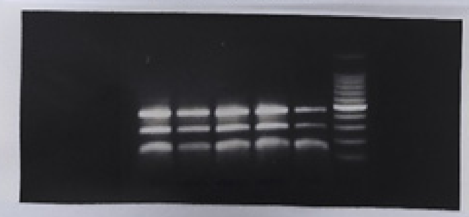

Analysis of the MSP-1 *Plasmodium vivax* Gene

Restriction Enzyme Reactions= Alu I

Reagents used	Quantities for reaction
Buffer 10x	2 µl
BSA 10 µg/ µl	0.2 µl
Enzyme Alu I	0.5 µl
DNA amplified product	5 µl
Nuclease-free water	12.3 µl
**Total**	20 µl
**Samples evaluated**	Results
Lane 1 (sample VE04)	Genotype 1
Lane 2 (sample VE05)	Genotype 1
Lane 3 (sample VE06)	Genotype 1
Lane 4 (sample VE07)	Genotype 1
Lane 4 (sample VE07)	Genotype 1
Lane 6 (molecular marker)	Promega DNA Ladder 100bp

1 2 3 4 5 6

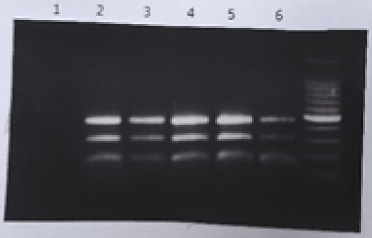

Analysis of the MSP-1 *Plasmodium vivax* Gene

Restriction Enzyme Reactions= Alu I

Reagents used	Quantities for reaction
Buffer 10x	2 µl
BSA 10 µg/ µl	0.2 µl
Enzyme Alu I	0.5 µl
DNA amplified product	5 µl
Nuclease-free water	12.3 µl
**Total**	20 µl
**Samples evaluated**	Results
Lane 1 (molecular marker)	Promega DNA Ladder 100bp
Lane 2 (sample BT19)	Genotype 1
Lane 3 (sample BT20)	Genotype 1
Lane 4 (sample BT21)	Genotype 1
Lane 5 (sample BT22)	Genotype 1
Lane 6 (sample BT23)	Genotype 1

1 2 3 4 5 6

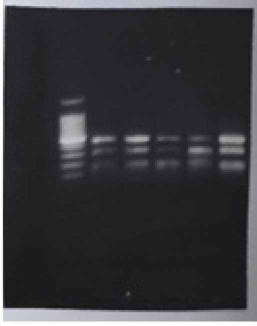

Analysis of the MSP-1 *Plasmodium vivax* Gene

Restriction Enzyme Reactions= Alu I

Reagents used	Quantities for reaction
Buffer 10x	2 µl
BSA 10 µg/ µl	0.2 µl
EnzymeAlu I	0.5 µl
DNA amplified product	5 µl
Nuclease-free water	12.3 µl
**Total**	20 µl
**Samples evaluated**	**Results**
Lane 1 (molecular marker)	Promega DNA Ladder 100bp
Lane 2 (sample PE008)	Genotype I
Lane 3 (sample PE009)	Genotype I
Lane 4 (sample PE010)	Genotype I
Lane 5 (sample PE011)	Genotype I
Lane 6 (sample PE012)	Genotype I

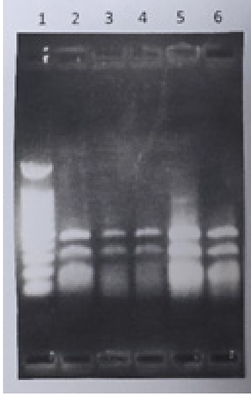

Analysis of the MSP-1 *Plasmodium vivax* Gene

Restriction Enzyme Reactions= Alu I

**Reagents used**	**Quantities for reaction**
Buffer 10x	2 µl
BSA 10 µg/ µl	0.2 µl
EnzymeAlu I	0.5 µl
DNA amplified product	5 µl
Nuclease-free water	12.3 µl
**Total**	20 µl
**Samples evaluated**	**Results**
Lane 1 (molecular marker)	Promega DNA Ladder 100bp
Lane 2 (sample VE013)	Genotype I
Lane 3 (sample VE014)	Genotype I
Lane 4 (sample VE015)	Genotype I
Lane 5 (sample VE016)	Genotype I
Lane 6 (sample VE017)	Genotype I

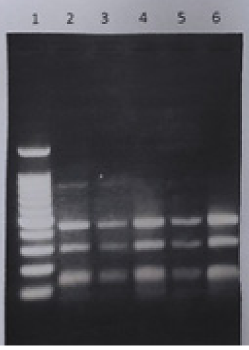

Analysis of the MSP-1 *Plasmodium vivax* Gene

Restriction Enzyme Reactions= Alu I

Reagents used	Quantities for reaction
Buffer 10x	2 µl
BSA 10 µg/ µl	0.2 µl
Enzyme Alu I	0.5 µl
DNA amplified product	5 µl
Nuclease-free water	12.3 µl
**Total**	20 µl
**Samples evaluated**	Results
Lane 1 (sample DA33)	Genotype 1
Lane 2 (sample DA34)	repeat sample
Lane 3 (sample DA35)	Genotype 1
Lane 4 (sample DA36)	Genotype 1
Lane 5 (sample DA37)	Genotype 1
Lane 6 (molecular marker)	Promega DNA Ladder 100bp

1 2 3 4 5 6

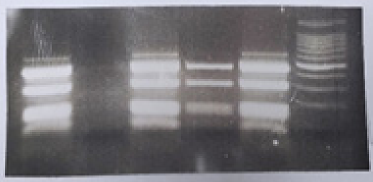

Analysis of the MSP-1 *Plasmodium vivax* Gene

Restriction Enzyme Reactions= Alu I

Reagents used	Quantities for reaction
Buffer 10x	2 µl
BSA 10 µg/ µl	0.2 µl
Enzyme Alu I	0.5 µl
DNA amplified product	5 µl
Nuclease-free water	12.3 µl
**Total**	20 µl
**Samples evaluated**	**Results**
Lane 1 (molecular marker)	Promega DNA Ladder 100bp
Lane 2 (sample PE42)	Genotype 1
Lane 3 (sample PE44)	Genotype 1
Lane 4 (sample PE48)	Genotype 1
Lane 5 (sample PE47)	Genotype 1
Lane 6 (sample PM02)	Genotype 1

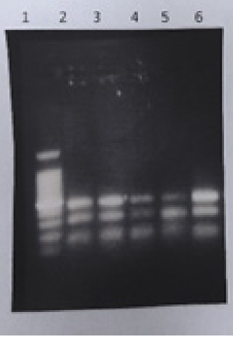

Analysis of the MSP-1 *Plasmodium vivax* Gene

Restriction Enzyme Reactions= Alu I

**Reagents used**	**Quantities for reaction**
Buffer 10x	2 µl
BSA 10 µg/ µl	0.2 µl
Enzyme Alu I	0.5 µl
DNA amplified product	5 µl
Nuclease-free water	12.3 µl
**Total**	20 µl
**Samples evaluated**	**Results**
Lane 1 (molecular marker)	Promega DNA Ladder 100bp
Lane 2 (sample PO43)	Genotype 2
Lane 3 (sample PO45)	Genotype 2
Lane 4 (sample PE43)	Genotype 1
Lane 4 (sample PO07)	Genotype 1

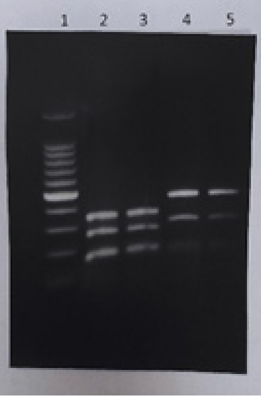

Analysis of the MSP-1 Plasmodium vivax Gene

Restriction Enzyme Reactions= Alu I

**Reagents used**	**Quantities for reaction**
Buffer 10x	2 µl
BSA 10 µg/ µl	0.2 µl
Enzyme Alu I	0.5 µl
DNA amplified product	5 µl
Nuclease-free water	12.3 µl
**Total**	20 µl
**Samples evaluated**	**Results**
Lane 1 (molecular marker)	Promega DNA Ladder 100bp
Lane 2 (sample VE25)	Genotype 1
Lane 3 (sample VE25)	Genotype 1
Lane 4 (sample PE48)	Genotype 1
Lane 5 (sample PE49)	Genotype 1
Lane 6 (sample PE52)	Genotype 1
Lane 7 (sample PE56)	Genotype 1
Lane 8 (sample PE53)	Genotype 1

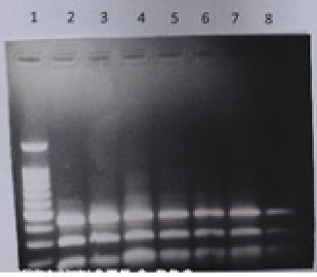

Analysis of the MSP-1 *Plasmodium vivax* Gene

Restriction Enzyme Reactions= Alu I

Reagents used	Quantities for reaction
Buffer 10x	2 µl
BSA 10 µg/ µl	0.2 µl
EnzymeAlu I	0.5 µl
DNA amplified product	5 µl
Nuclease-free water	12.3 µl
**Total**	20 µl
**Samples evaluated**	**Results**
Lane 1 (sample DA51)	Genotype 2
Lane 2 (sample DA50)	Genotype 1
Lane 3 undigested secondary PCR product	undigested product
Lane 4 undigested secondary PCR product	undigested product
Lane 5 (molecular marker)	Promega DNA Ladder 100bp

1 2 3 4 5

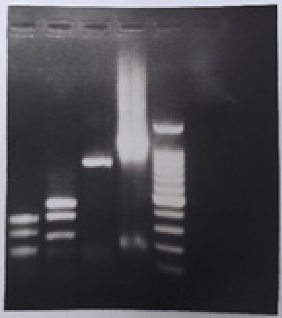

Supplement: Supplementary file 1 [file pathogens-10-00989-s001.zip › Figure S2 Msp-1 -AluI profiles.pdf]
