# Supplementary material for: Plasmodium vivax Genetic Diversity in Panama: Challenges for Malaria Elimination in Mesoamerica
Source: Pathogens. 2021 Aug 5;10(8):989. doi: 10.3390/pathogens10080989 (PMC8401434; doi:10.3390/pathogens10080989)

Figure S3. PCR-RFLP profiles of *msp-3α* gene amplified from *P. vivax* study samples digested with *Hha* I enzyme.

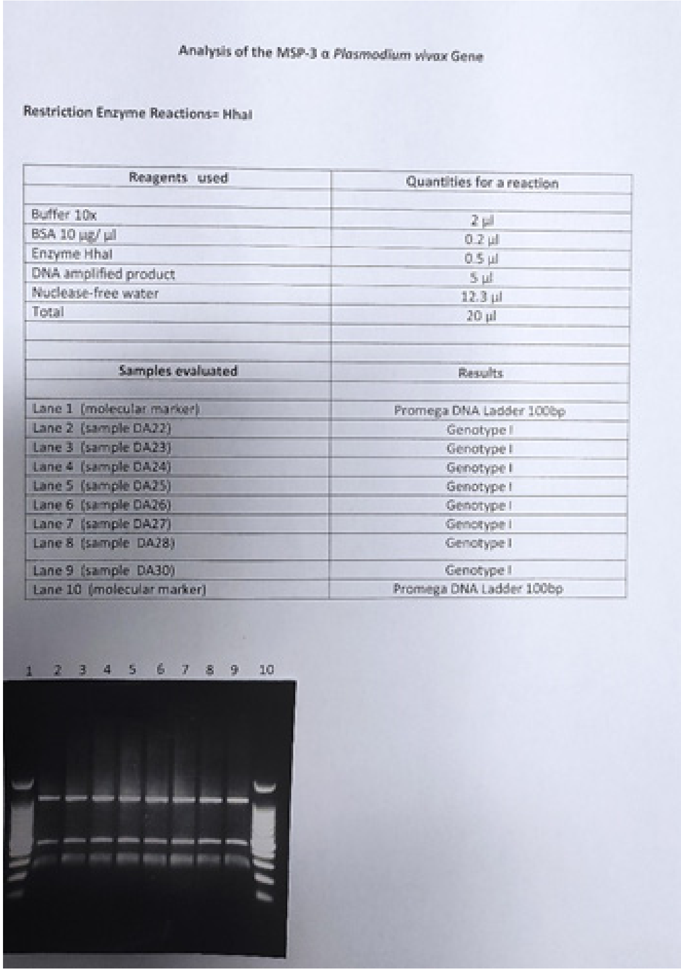

Analysis of the MSP-3 α *Plasmodium vivax* Gene

Restriction Enzyme Reactions= HhaI

Reagents used	Quantities for a reaction
Buffer 10x	2 µl
BSA 10 µg/ µl	0.2 µl
Enzyme HhaI	0.5 µl
DNA amplified product	5 µl
Nuclease-free water	12.3 µl
Total	20 µl
**Samples evaluated**	Results
Lane 1 (molecular marker)	Promega DNA Ladder 100bp
Lane 2 (sample DA22)	Genotype I
Lane 3 (sample DA23)	Genotype I
Lane 4 (sample DA24)	Genotype I
Lane 5 (sample DA25)	Genotype I
Lane 6 (sample DA26)	Genotype I
Lane 7 (sample DA27)	Genotype I
Lane 8 (sample DA28)	Genotype I
Lane 9 (sample DA30)	Genotype I
Lane 10 (molecular marker)	Promega DNA Ladder 100bp

Analysis of the MSP-3 α *Plasmodium vivax* Gene

Restriction Enzyme Reactions= HhaI

Reagents used	Quantities for a reaction
Buffer 10x	2 µl
BSA 10 µg/ µl	0.2 µl
Enzyme HhaI	0.5 µl
DNA amplified product	5 µl
Nuclease-free water	12.3 µl
**Total**	20 µl
**Samples evaluated**	Results
Lane 1 (molecular marker)	Promega DNA Ladder 100bp
Lane 2 (sample DA65)	Genotype 2
Lane 3 (sample DA66)	Genotype 2
Lane 4 (sample DA67)	Genotype 2
Lane 5 (sampleDA68)	Genotype 2
Lane 6 (sample DA69)	Genotype 2
Lane 7 (sample DA71)	Genotype 2
Lane 8 (sample DA73)	Genotype I
Lane 9 undigested secondary PCR product	undigested product

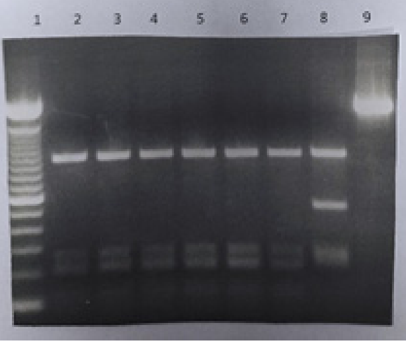

Analysis of the MSP-3 α *Plasmodium vivax* Gene

Restriction Enzyme Reactions= HhaI

Reagents used	Quantities for a reaction
Buffer 10x	2 µl
BSA 10 µg/ µl	0.2 µl
Enzyme HhaI	0.5 µl
DNA amplified product	5 µl
Nuclease-free water	12.3 µl
**Total**	20 µl
**Samples evaluated**	**Results**
Lane 1 (molecular marker)	Promega DNA Ladder 100bp
Lane 2 (sample PE51)	Genotype 2
Lane 3 (sample PE59)	repeat sample
Lane 4 (sample DA34)	Genotype 2
Lane 5 (sample DA38)	Genotype 2
Lane 6 (sample DA43)	Genotype 2
Lane 7 (sample DA45)	Genotype 1
Lane 8 (sample DA46)	Genotype 2
Lane 9 (sample DA48)	Genotype 2
Lane 10 (sample DA53)	Genotype 2
Lane 11 (sample PO01)	Genotype 3
Lane 12 (sample KY151)	Genotype 2
Lane 13 (sample DA52)	Genotype 2
Lane 14 (sample DA54)	Genotype 2

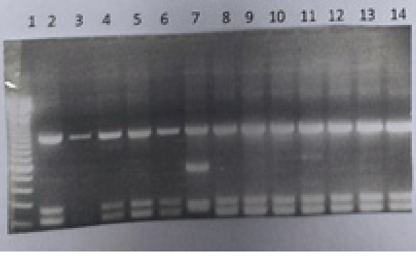

Analysis of the MSP-3 α *Plasmodium vivax* Gene

Restriction Enzyme Reactions= HhaI

**Reagents used**	**Quantities for a reaction**
Buffer 10x	2 µl
BSA 10 µg/ µl	0.2 µl
Enzyme HhaI	0.5 µl
DNA amplified product	5 µl
Nuclease-free water	12.3 µl
**Total**	20 µl
**Samples evaluated**	**Results**
Lane 1 (sample PO01)	Genotype 3
Lane 2 (sample DA49)	Genotype I
Lane 3 (sample DA52)	Genotype 2
Lane 4 (sample DA53)	Genotype 2
Lane 5 (sample DA40)	Genotype 2
Lane 6 (sample DA38)	Genotype 2
Lane 7 (sample DA43)	Genotype 2
Lane 8 (molecular marker)	Promega DNA Ladder 100bp

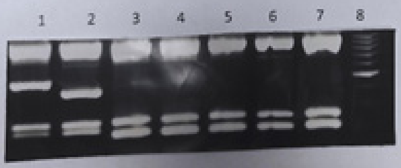

Analysis of the MSP-3 α *Plasmodium vivax* Gene

Restriction Enzyme Reactions= HhaI

**Reagents used**	**Quantities for a reaction**
Buffer 10x	2 µl
BSA 10 µg/ µl	0.2 µl
Enzyme HhaI	0.5 µl
DNA amplified product	5 µl
Nuclease-free water	12.3 µl
**Total**	20 µl
**Samples evaluated**	**Results**
Lane 1 (molecular marker)	Promega DNA Ladder 100bp
Lane 2 (sample KY154)	Genotype 2
Lane 3 (sample KY155)	Genotype 2
Lane 4 (sample KY140)	Genotype I
Lane 5 (sample KY141)	Genotype I
Lane 6 (sample KY143)	Genotype I
Lane 7 (sample KY130)	Genotype 2
Lane 8 (sample KY131)	Genotype 2
Lane 9 (sample KY134)	Genotype 2
Lane 10 (sample KY150)	Genotype 2

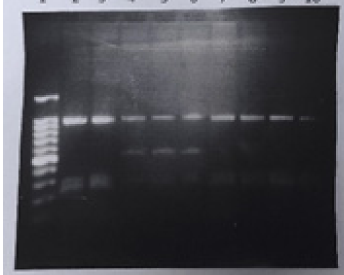

Analysis of the MSP-3 α *Plasmodium vivax* Gene

Restriction Enzyme Reactions= HhaI

Reagents used	Quantities for a reaction
Buffer 10x	2 µl
BSA 10 µg/ µl	0.2 µl
Enzyme HhaI	0.5 µl
DNA amplified product	5 µl
Nuclease-free water	12.3 µl
Total	20 µl
**Samples evaluated**	Results
Lane 1 (molecular marker)	Promega DNA Ladder 100bp
Lane 2 (sample PO37)	Genotype I
Lane 3 (sample PO36)	Genotype I
Lane 4 (sample PO35)	Genotype I
Lane 5 (sample PO34)	Genotype I
Lane 6 (sample PO33)	Genotype I
Lane 7 (sample PO32)	Genotype I
Lane 8 (sample PO31)	Genotype I
Lane 9 (sample PO30)	Genotype I
Lane 10 (sample PO29)	Genotype I

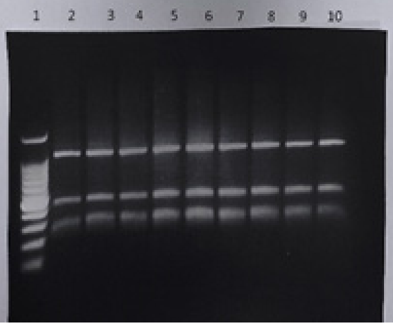

Analysis of the MSP-3 α *Plasmodium vivax* Gene

Restriction Enzyme Reactions= HhaI

Reagents used	Quantities for a reaction
Buffer 10x	2 µl
BSA 10 µg/ µl	0.2 µl
Enzyme HhaI	0.5 µl
DNA amplified product	5 µl
Nuclease-free water	12.3 µl
**Total**	20 µl
**Samples evaluated**	**Results**
Lane 1 (molecular marker)	Promega DNA Ladder 100bp
Lane 2 (sample PE05)	Genotype 1
Lane 3 (sample PE06)	Genotype 1
Lane 4 (sample PE07)	Genotype 1
Lane 5 (sample PE08)	Genotype 1
Lane 6 (sample PE09)	Genotype 1
Lane 7 (sample PE10)	Genotype 1
Lane 8 (sample PE11)	Genotype 1
Lane 9 (sample PE12)	Genotype 1
Lane 10 (sample PE13)	Genotype 1

1 2 3 4 5 6 7 8 9 10

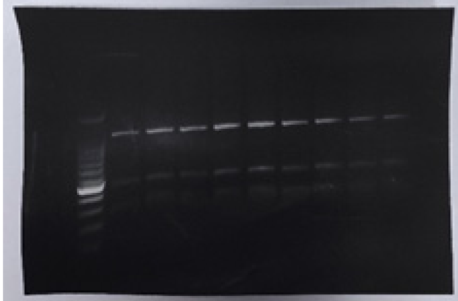

Analysis of the MSP-3 α *Plasmodium vivax* Gene

Restriction Enzyme Reactions= HhaI

Reagents used	Quantities for a reaction
Buffer 10x	2 µl
BSA 10 µg/ µl	0.2 µl
Enzyme HhaI	0.5 µl
DNA amplified product	5 µl
Nuclease-free water	12.3 µl
Total	20 µl
**Samples evaluated**	**Results**
Lane 1 (sample DA45)	Genotype 2
Lane 2 (sample PE51)	Genotype 2
Lane 3 (sample PE59)	Genotype 2
Lane 4 (sample DA34)	Genotype 2
Lane 5 (sample DA18)	Genotype 2
Lane 6 (sample DA01)	Genotype 2
Lane 7 (molecular marker)	Promega DNA Ladder 100bp

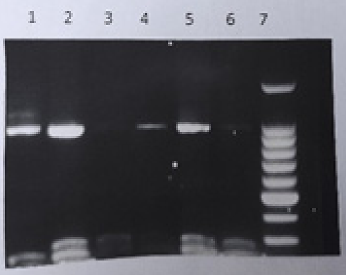

Analysis of the MSP-1 *Plasmodium vivax* Gene

Restriction Enzyme Reactions= Alu I

Reagents used	Quantities for reaction
Buffer 10x	2 µl
BSA 10 µg/ µl	0.2 µl
Enzyme Alu I	0.5 µl
DNA amplified product	5 µl
Nuclease-free water	12.3 µl
**Total**	20 µl
**Samples evaluated**	Results
Lane 1 (molecular marker)	Promega DNA Ladder 100bp
Lane 2 (sample PE34)	Genotype 1
Lane 3 (sample PE35)	Genotype 1
Lane 4 (sample PE36)	Genotype 1
Lane 5 (sample BT08)	Genotype 1
Lane 6 (sample BT12)	Genotype 1
Lane 7 (sample BT13)	Genotype 1
Lane 8 (sample DA06)	Genotype 1
Lane 9 (sample DA08)	Genotype 1
Lane 10 (simple DA09)	Genotype 1
Lane 11 (sample DA14)	Genotype 1
Lane 12 undigested secondary PCR product	undigested product

1 2 3 4 5 6 7 8 9 10 11 12

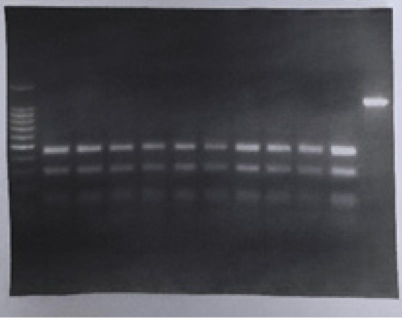

Analysis of the MSP-3 α *Plasmodium vivax* Gene

Restriction Enzyme Reactions= HhaI

**Reagents used**	**Quantities for a reaction**
Buffer 10x	2 µl
BSA 10 µg/ µl	0.2 µl
Enzyme HhaI	0.5 µl
DNA amplified product	5 µl
Nuclease-free water	12.3 µl
Total	20 µl
**Samples evaluated**	**Results**
Lane 1 (molecular marker)	Promega DNA Ladder 100bp
Lane 2 (sample PO01)	Repeat sample
Lane 3 (sample DA34)	Repeat sample
Lane 4 (sample DA38)	Genotype 2
Lane 5 (sample DA43)	Genotype 2
Lane 6 (sample DA46)	Genotype 2
Lane 7 (sample DA45)	Genotype I
Lane 8 (sample KY151)	Genotype 2
Lane 9 (sample DA52)	Genotype 2
Lane 10 (sample DA54)	Genotype 2

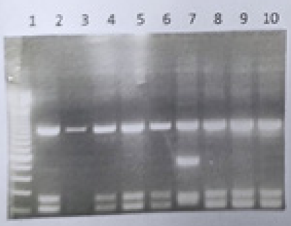

Analysis of the MSP-1 *Plasmodium vivax* Gene

Restriction Enzyme Reactions= Alu I

**Reagents used**	**Quantities for reaction**
Buffer 10x	2 µl
BSA 10 µg/ µl	0.2 µl
Enzyme Alu I	0.5 µl
DNA amplified product	5 µl
Nuclease-free water	12.3 µl
**Total**	20 µl
**Samples evaluated**	Results
Lane 1 (molecular marker)	Promega DNA Ladder 100bp
Lane 2 (sample PE60)	Genotype 2
Lane 3 (sample PE58)	Genotype 2
Lane 4 (sample PE65)	Genotype 1
Lane 5 (sample BT16)	Genotype 1
Lane 6 (simple DA25)	Genotype 1

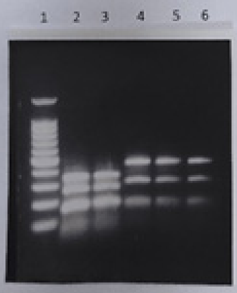

Analysis of the MSP-1 *Plasmodium vivax* Gene

Restriction Enzyme Reactions= Alu I

Reagents used	Quantities for reaction
Buffer 10x	2 µl
BSA 10 µg/ µl	0.2 µl
Enzyme Alu I	0.5 µl
DNA amplified product	5 µl
Nuclease-free water	12.3 µl
**Total**	20 µl
**Samples evaluated**	Results
Lane 1 (sample PE037)	Genotype 1
Lane 2 (simple PE38)	Genotype 1
Lane 3 (sample PE39)	Genotype 1
Lane 4 (sample PE40)	Genotype 1
Lane 5 (sample PE41)	Genotype 1
Lane 6 (molecular marker)	Promega DNA Ladder 100bp

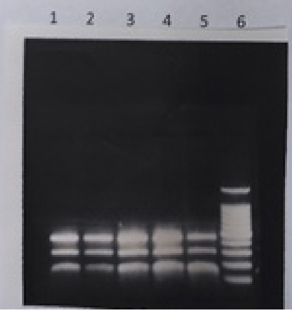

Analysis of the MSP-3 α *Plasmodium vivax* Gene

Restriction Enzyme Reactions= HhaI

Reagents used	Quantities for a reaction
Buffer 10x	2 µl
BSA 10 µg/ µl	0.2 µl
Enzyme HhaI	0.5 µl
DNA amplified product	5 µl
Nuclease-free water	12.3 µl
Total	20 µl
**Samples evaluated**	Results
Lane 1 (simple PO38)	Genotype I
Lane 2 (sample PO39)	repeat sample
Lane 3 (sample PO42)	Genotype I
Lane 4 (sample PO43)	repeat sample
Lane 5 (sample PO44)	repeat sample
Lane 6 (sample PO49)	repeat sample
Lane 7 (sample PO54)	repeat sample
Lane 8 (sample PO56)	repeat sample
Lane 9 (sample PO59)	repeat sample
Lane 10 (molecular marker)	Promega DNA Ladder 100bp

1 2 3 4 5 6 7 8 9 10

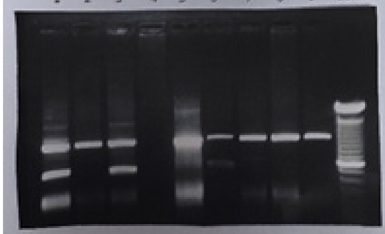

Analysis of the MSP-3 α *Plasmodium vivax* Gene

Restriction Enzyme Reactions= HhaI

Reagents used	Quantities for a reaction
Buffer 10x	2 µl
BSA 10 µg/ µl	0.2 µl
Enzyme HhaI	0.5 µl
DNA amplified product	5 µl
Nuclease-free water	12.3 µl
Total	20 µl
**Samples evaluated**	Results
Lane 1 (sample PE51)	Genotype 2
Lane 2 (sample DA46)	Genotype 2
Lane 3 (sample PE41)	Genotype 1
Lane 4 (sample DA20)	Genotype 3
Lane 5 (molecular marker)	Promega DNA Ladder 100 bp

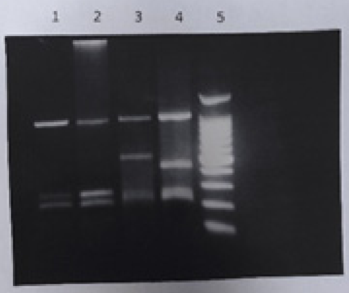

Analysis of the MSP-3 α *Plasmodium vivax* Gene

Restriction Enzyme Reactions= HhaI

Reagents used	Quantities for a reaction
Buffer 10x	2 µl
BSA 10 µg/ µl	0.2 µl
Enzyme HhaI	0.5 µl
DNA amplified product	5 µl
Nuclease-free water	12.3 µl
Total	20 µl
**Samples evaluated**	**Results**
Lane 1 (sample PE59)	Genotype 2
Lane 2 (sample DA45)	Genotype 2
Lane 3 (sample PE59)	Genotype 2
Lane 4 (sample PE40)	Genotype 1
Lane 5 (molecular marker)	Promega DNA Ladder 100 bp

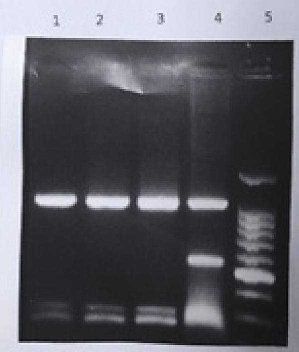

Analysis of the MSP-3 α *Plasmodium vivax* Gene

Restriction Enzyme Reactions= HhaI

**Reagents used**	**Quantities for a reaction**
Buffer 10x	2 µl
BSA 10 µg/ µl	0.2 µl
Enzyme HhaI	0.5 µl
DNA amplified product	5 µl
Nuclease-free water	12.3 µl
Total	20 µl
**Samples evaluated**	**Results**
Lane 1 (molecular marker)	Promega DNA Ladder 100bp
Lane 2 (sample DA22)	Genotype I
Lane 3 (sample DA23)	Genotype I
Lane 4 (sample DA24)	Genotype I
Lane 5 (sample DA25)	repeat sample
Lane 6 (sample DA26)	repeat sample
Lane 7 (sample DA27)	Genotype I
Lane 8 (sample DA28)	repeat sample
Lane 9 (sample DA30)	repeat sample
Lane 10 (sample DA30)	Genotype I

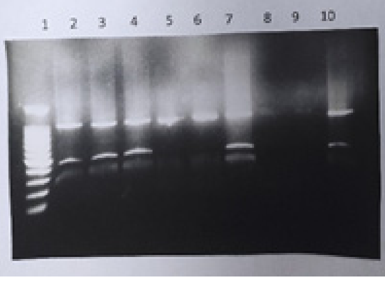

Analysis of the MSP-3 α *Plasmodium vivax* Gene

Restriction Enzyme Reactions= HhaI

**Reagents used**	Quantities for a reaction
Buffer 10x	2 µl
BSA 10 µg/ µl	0.2 µl
Enzima HhaI	0.5 µl
DNA (producto amplificado II PCR)	5 µl
Agua libre de nucleasas	12.3 µl
**Total por reacción**	20 µl
**Samples evaluated**	**Results**
Lane 1 (sample VE19)	Genotype I
Lane 2 (sample VE20)	Genotype I
Lane 3 (sample VE21)	repeat sample
Lane 4 (sample DA43)	repeat sample
Lane 5 (sample DA44)	Genotype 2
Lane 5 (sample DA44)	Genotype 2
Lane 6 (molecular marker)	Promega DNA Ladder 1Kb

1 2 3 4 5 6 7

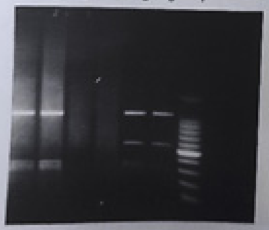

Analysis of the MSP-3 α *Plasmodium vivax* Gene

Restriction Enzyme Reactions= HhaI

Reagents used	Quantities for a reaction
Buffer 10x	2 µl
BSA 10 µg/ µl	0.2 µl
Enzyme HhaI	0.5 µl
DNA amplified product	5 µl
Nuclease-free water	12.3 µl
Total	20 µl
**Samples evaluated**	**Results**
Lane 1 (sample VE12)	Genotype I
Lane 2 (sample VE13)	Genotype I
Lane 3 (sample VE14)	Genotype I
Lane 4 (sample VE15)	Genotype I
Lane 5 (sample VE19)	repeat sample
Lane 5 (molecular marker)	Promega DNA Ladder 100bp

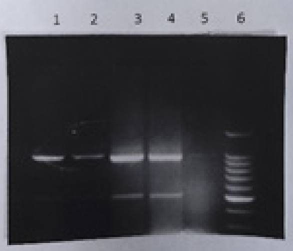

Analysis of the MSP-3 α *Plasmodium vivax* Gene

Restriction Enzyme Reactions= HhaI

Reagents used	Quantities for a reaction
Buffer 10x	
BSA 10 µg/ µl	2 µl
Enzyme HhaI	0.2 µl
DNA amplified product	0.5 µl
Nuclease-free water	5 µl
Total	12.3 µl
	20 µl
**Samples evaluated**	**Results**
Lane 1 (molecular marker)	Promega DNA Ladder 100bp
Lane 2 (sample DOBA01)	Genotype I
Lane 3 (sample DOBA02)	Genotype I
Lane 4 (sample DOBA03)	repeat sample
Lane 5 (sample DOBA04)	repeat sample
Lane 6 (sample DOBA05)	Genotype I
Lane 7 (sample DOBA07)	Genotype I

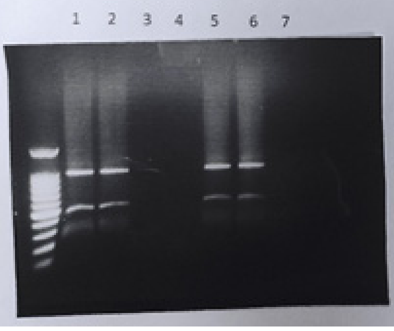

Analysis of the MSP-3 α *Plasmodium vivax* Gene

Restriction Enzyme Reactions= HhaI

Reagents used	Quantities for a reaction
Buffer 10x	2 µl
BSA 10 µg/ µl	0.2 µl
Enzyme HhaI	0.5 µl
DNA amplified product	5 µl
Nuclease-free water	12.3 µl
Total	20 µl
**Samples evaluated**	**Results**
Lane 1 (molecular marker)	Promega DNA Ladder 100bp
Lane 2 (sample PE59)	Repeat sample
Lane 3 (sample DA16)	Repeat sample
Lane 4 (PO23)	Genotype 1
Lane 5 (PO24)	Genotype 1

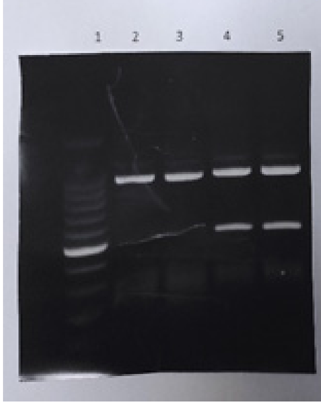

Analysis of the MSP-3 α *Plasmodium vivax* Gene

Restriction Enzyme Reactions= HhaI

Reagents used	Quantities for a reaction
Buffer 10x	2 µl
BSA 10 µg/ µl	0.2 µl
Enzyme HhaI	0.5 µl
DNA amplified product	5 µl
Nuclease-free water	12.3 µl
**Total**	20 µl
**Samples evaluated**	Results
Lane 1 (molecular marker)	Promega DNA Ladder 100bp
Lane 2 (sample DA54)	Genotype 2
Lane 3 (sample PO61)	Genotype 2
Lane 4 (sample PO62)	Genotype 2
Lane 5 (sample PO50)	Genotype 2
Lane 6 (sample PO40)	Genotype 2
Lane 7 (sample PO07)	Genotype 2
Lane 8 (sample KY165)	Genotype 2
Lane 9 (sample KY166)	Genotype 2
Lane 10 (sample KY159)	Genotype 2
Lane 11 (sample KY161)	Genotype 2
Lane 12 (sample KY151)	Genotype 2
Lane 13 (sample DA52)	Genotype 2
Lane 14 (sample DA54)	Genotype 2

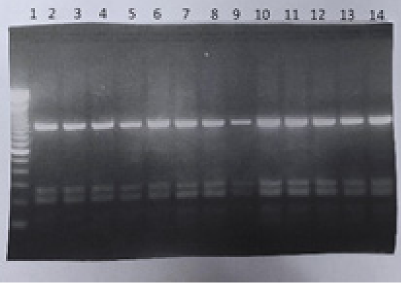

Analysis of the MSP-3 α *Plasmodium vivax* Gene

Restriction Enzyme Reactions= HhaI

Reagents used	Quantities for a reaction
Buffer 10x	2 µl
BSA 10 µg/ µl	0.2 µl
Enzyme HhaI	0.5 µl
DNA amplified product	5 µl
Nuclease-free water	12.3 µl
Total	20 µl
**Samples evaluated**	Results
Lane 1 (sample VE08)	repeat sample
Lane 2 (sample VE09)	repeat sample
Lane 3 (sample VE10)	repeat sample
Lane 4 (sample VE11)	Genotype 1
Lane 5 (sample VE12)	repeat sample
Lane 6 (molecular marker)	Promega DNA Ladder 100bp

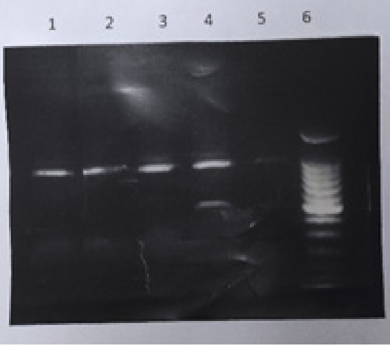

Analysis of the MSP-3 α *Plasmodium vivax* Gene

Restriction Enzyme Reactions= HhaI

Reagents used	Quantities for a reaction
Buffer 10x	2 µl
BSA 10 µg/ µl	0.2 µl
Enzyme HhaI	0.5 µl
DNA amplified product	5 µl
Nuclease-free water	12.3 µl
**Total**	20 µl
**Samples evaluated**	**Results**
Lane 1 (molecular marker)	Promega DNA Ladder 100bp
Lane 2 (sample DA01)	Genotype 2
Lane 3 (sample DA06)	Genotype 2
Lane 4 (sample DA08)	Genotype 2
Lane 5 (sampleDA09)	Genotype 2
Lane 6 (sample DA10)	Genotype 2
Lane 7 (sample Da16)	Genotype 2
Lane 8 (sample Da18)	Genotype 2
Lane 9 (sample DA19)	Genotype 2
Lane 10 (sample DA31)	Genotype 2

1 2 3 4 5 6 7 8 9 10

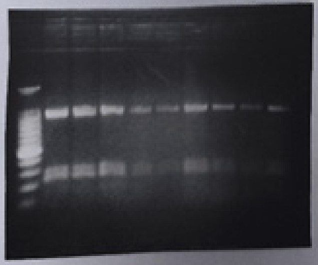

Analysis of the MSP-3 α *Plasmodium vivax* Gene

Restriction Enzyme Reactions= HhaI

Reagents used	Quantities for a reaction
Buffer 10x	2 µl
BSA 10 µg/ µl	0.2 µl
Enzyme HhaI	0.5 µl
DNA amplified product	5 µl
Nuclease-free water	12.3 µl
Total	20 µl
**Samples evaluated**	**Results**
Lane 1 (sample DA15)	Genotype 1
Lane 2 (sample DA17)	Genotype 1
Lane 3 (sample VE10)	Genotype 1
Lane 4 (sample VE12)	repeat sample
Lane 5 (sample VE14)	repeat sample
Lane 6 (molecular marker)	Promega DNA Ladder 100bp

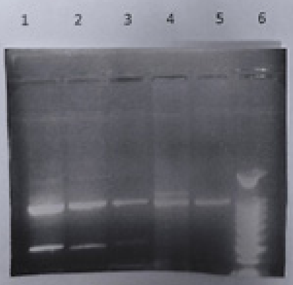

Supplement: Supplementary file 1 [file pathogens-10-00989-s001.zip › Figure S3 Msp-3 -Hha I profiles.pdf]
